# Deep mRNA sequencing reveals stage-specific transcriptome alterations during microsclerotia development in the smoke tree vascular wilt pathogen, *Verticillium dahliae*

**DOI:** 10.1186/1471-2164-15-324

**Published:** 2014-05-01

**Authors:** Dianguang Xiong, Yonglin Wang, Jie Ma, Steven J Klosterman, Shuxiao Xiao, Chengming Tian

**Affiliations:** The Key Laboratory for Silviculture and Conservation of Ministry of Education, College of Forestry, Beijing Forestry University, Beijing, China; School of Information Science and Technology, Beijing Forestry University, Beijing, China; United States Department of Agriculture-Agricultural Research Service, Salinas, CA USA

**Keywords:** *Verticillium dahliae*, Microsclerotia development, RNA-Seq, Transcriptome, Gene expression, Alternative splicing

## Abstract

**Background:**

*Verticillium dahliae* is a soil-borne fungus that causes vascular wilt diseases in a wide range of plant hosts. *V. dahliae* produces multicelled, melanized resting bodies, also known as microsclerotia (MS) that can survive for years in the soil. The MS are the primary source of infection of the Verticillium disease cycle. Thus, MS formation marks an important event in the disease cycle of *V. dahliae*.

**Results:**

In this study, next generation sequencing technology of RNA-Seq was employed to investigate the global transcriptomic dynamics of MS development to identify differential gene expression at several stages of MS formation in strain XS11 of *V. dahliae,* isolated from smoke tree. We observed large-scale changes in gene expression during MS formation, such as increased expression of genes involved in protein metabolism and carbohydrate metabolism. Genes involved in glycolytic pathway and melanin biosynthesis were dramatically up-regulated in MS. Cluster analyses revealed increased expression of genes encoding products involved in primary metabolism and stress responses throughout MS development. Differential expression of ubiquitin-dependent protein catabolism and cell death-associated genes during MS development were revealed. Homologs of genes located in the lineage-specific (LS) regions of *V. dahliae* strain VdLs.17, were either not expressed or showed low expression. Furthermore, alternative splicing (AS) events were analyzed, revealing that over 95.0% AS events involve retention of introns (RI).

**Conclusions:**

These data reveal the dynamics of transcriptional regulation during MS formation and were used to construct a comprehensive high-resolution gene expression map. This map provides a key resource for understanding the biology and molecular basis of MS development of *V. dahliae.*

**Electronic supplementary material:**

The online version of this article (doi:10.1186/1471-2164-15-324) contains supplementary material, which is available to authorized users.

## Background

*Verticillium dahliae* Kleb. (Eukaryota, Fungi, Ascomycota) is a ubiquitous soil-borne fungus that penetrates plant roots, enters the plant vascular system, and causes vascular wilt diseases collectively known as Verticillium wilts [1, 2]. *V. dahliae* can infect more than 200 plant species, including important crops, flowers, vegetables, trees, and shrubs, causing economically significant losses each year [1, 2].

Verticillium wilt is a threat to smoke tree (*Cotinus coggygria* Scop.) stands in China. Smoke trees are of primary importance as ornamentals, highly valued for the brilliant red leaf scenery that these trees provide in the Beijing region during autumn, especially in Fragrant Hills Park, a 160 hectare forest park in Beijing. Vascular wilt in smoke tree was reported in Fragrant Hills Park as early as 1990, and subsequently the disease spread to other smoke tree growing areas in Beijing [3]. Symptoms of vascular wilt in smoke trees include stunted growth of the stem, early senescence of leaves, and early mortality. Without adequate control, the famous “red leaf scenery” of Fragrant Hills and other areas is threatened by the detrimental effects of the Verticillium wilt. Currently available fungicides and other control measures are not effective in controlling the disease due in part to the ability of the fungus to survive for long periods in soil.

The survival of *V. dahliae* in soil depends on the production of melanized, multicellular structures known as microsclerotia (MS) [4]. Melanin deposition and a thickened cell wall enable the MS of the pathogen to resist UV irradiation, temperature extremes, enzymatic lysis, and fungicidal activities [5]. The MS can survive in soils in the absence of a host plant for as long as 10 years, and are the primary infectious propagules of the Verticillium wilt disease [6]. The MS germinate to form hyphae in the soil, and penetrate the plant roots, where the fungus colonizes the xylem tissue of the plant vascular system. As wilt symptoms progress, *V. dahliae* produces MS in dying plant tissues, which are returned to the soil to initiate new primary infections. Thus, the production of MS represents a significant developmental event in the life cycle of *V. dahliae*[7].

The morphological events of MS formation have been well studied by both light and electron microscopy [8–11]. In the early stages of MS development, hyphae become swollen, vacuolated, and form numerous septa. Subsequently, clusters of hyphal cells form in the swollen hyphae that resemble the microsclerotial initial [11, 12]. In the final phase of MS formation, melanin particles are extruded into the interhyphal spaces of the microsclerotium, and peripheral microsclerotial cells are killed by autolysis [8]. The genes involved in melanin biosynthesis have been identified and their functions have been characterized [10, 13–15]. The results indicate that melanin is necessary for the formation of fully functional MS [5]. However, little is known about the molecular pathways involved in MS formation.

Genome-wide identification of genes expressed during MS formation is a first step in elucidating the pathways and molecular mechanisms underlying MS formation in *V. dahliae*. Methods for genome-wide expression analyses include expressed sequence tag (EST) analysis [16, 17], suppression subtractive hybridization (SSH) [18, 19], serial analysis of gene expression (SAGE) [20], massive parallel signature sequencing (MPSS) [21–23] and RNA-Sequencing (RNA-Seq) [24–26]. Next-generation sequencing (NGS) technologies have provided new platforms for comprehensive transcriptional studies [27–31]. Transcriptome sequencing is an efficient means to generate transcriptomic data, and RNA-Seq is one approach transcriptome profiling that provides highly accurate measurements of gene expression by counting the number of sequencing reads, which map to a genome or annotated transcripts [28, 32], and further enable genome-wide identification of coding sequences, gene structure, alternative splicing [33]. Transcriptomic data produced by RNA-Seq methods have increased our understanding of gene expression involved in growth and development of pathogenic fungi [30, 34–39]. For example, transcriptional analysis of appressorium formation in the rice blast fungus *Magnaporthe oryzae* revealed the role of autophagy, lipid metabolism and melanin biosynthesis, and a Pmk1 MAPK kinase as a key global regulator in appressorium differentiation [39].

Resources available to facilitate transcriptome analyses of MS formation include transcript data for 10,535 genes and the 33.8 MB genome sequence for *V. dahliae*, strain VdLs.17 (Broad Institute Verticillium Group Database) [40]. Subsequent characterizations of this sequence resource also provide useful information to place transcriptomic studies in context. For example, comparison of the genome structure of *V. dahliae* strain VdLs.17 to that of the *Verticillium alfalfae* strain VaMs.102 revealed four lineage-specific (LS) regions of about 350 kb in length present in VdLs.17 but absent in the VaMs.102 strain. The VdLs.17 LS regions encoded 354 predicted genes, some of which had been associated with virulence and host range specificity. Only about 7.0% the LS genes encode predicted secreted proteins, and the LS regions were nearly devoid of “housekeeping” type genes [40]. Additional comparative genomics analyses of multiple *V. dahliae* strains indicated that the LS regions are diverse in length and gene content, enriched for *in planta*-expressed genes, and that chromosomal rearrangements associated with LS regions in *V. dahliae* are common [41]. An additional valuable resource includes expressed sequence tag (EST) libraries previously used to identify expressed genes in *V. dahliae* during pathogenic growth and MS development in *V. dahliae*[42]. Neumann and Dobinson [42] obtained about 1000 ESTs, many of which corresponded to melanin biosynthetic enzymes, exclusive to the developing MS culture type. The analysis of genes associated with specific ESTs has accelerated molecular characterization of MS formation. Recently, Duressa et al. performed a RNA-Seq analysis between MS producing cultures and those not producing MS in *V. dahliae*, revealing over 200 significantly expressed genes involved in melanin synthesis and other processes [43]. While this work focused on up- or down-regulation of gene expression between these two culture types, the focus was not on assessing gene expression associated with gradual developmental changes during MS formation.

In addition to those resources, several individual genes involved in MS formation have been characterized in *V. dahliae*[12, 44–47]. *VDH1*, encoding a class II hydrophobin, is one of the many genes significantly differentially expressed during MS formation, and *VDH1* is required for MS formation of *V. dahliae*[12, 45]. *VMK1*, encoding a mitogen-activated protein kinase, modulates MS formation [46]; mutation of *VdGARP1*, encoding a glutamic acid-rich protein, significantly delays development of melanized MS [44]. The non-LS copy of *VdHOG1*, a homolog of the high osmolarity glycerol response protein kinase, positively regulates MS formation (Xiao, et al., unpublished data), and the G protein β subunit negatively controls MS formation [47]. Functional characterizations of these individual genes have provided valuable insight into the genetic control of MS formation, yet there remain major gaps in our understanding of the molecular determinants that trigger and regulate MS formation in *V. dahliae*.

The objectives of this work were to (1) examine developmental stage-specific gene expression during MS formation in *V. dahliae* and (2) to identify genes differentially expressed in this developmental process. To accomplish these aims, extensive microscopy analyses were carried out to initially characterize four different stages of MS development in a smoke tree strain of *V. dahliae*. RNA-Seq analyses were employed to analyze transcript profiles of 10,158 genes (96.4% of the total predicted reference genes of the VdLs.17 strain) and global patterns of gene expression in *V. dahliae* using four identified MS developmental stages as reference points*.* This enabled identification genes significantly differentially expressed during stages of MS development, revealing major metabolic processes and signal pathways associated with MS development in *V. dahliae*. Elucidation of the molecular mechanisms that govern MS formation in *V. dahliae* may be useful in designing novel strategies to control Verticillium wilt, not only for the smoke tree pathogen, but also related pathogenic strains and other *Verticillium* spp. that produce MS [48].

## Results

### Microscopic analyses of MS development

**Figure 1:**
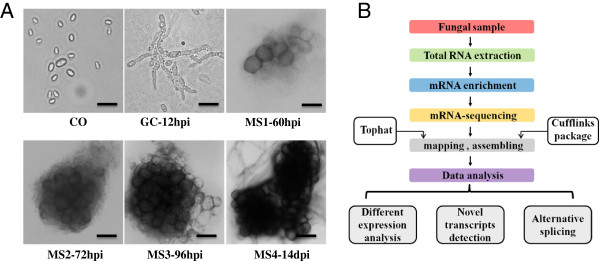
**Microsclerotia developmental process of the smoke tree vascular wilt fungus,** ***V. dahliae*** **. and the transcriptome analysis pipeline. A**. Micrographs showing microsclerotial development processes from conidia to microsclerotia in *Verticillium dahliae*. The panels represent different developmental stages of smoke tree wilt fungus cultured in CM broth and BM, respectively. Scale bar = 10 μm. MS1-MS4 represent four typical stages during the entire process of microsclerotia formation at 60 h, 72 h, 96 h and 14 d represent four stages of microsclerotia formation used for RNA-Seq. **B**. Flowcharts of the RNA-Seq method employed in this study. Tophat was used to align RNA-Seq reads to the genome of *V. dahliae* VdLs.17 (Broad Institute) and Cufflinks package was used to assemble and find differentially expressed genes and transcripts.

The dynamics of morphogenesis and pigment production were examined by light microscopy during MS development to chart cellular changes relative to gene expression during MS formation. Six stages were selected for these analyses based on phenotypic changes that were observed during MS formation. The stages of MS formation, as determined by light microscopy, were named MS1, MS2, MS3 and MS4 (Figure 1A). At 60 hpi (hours post incubation) or at MS1, cell clustering and swelling resembling the microsclerotial initial was observed at the margins of the cellulose membrane. This initial stage of MS development (60 hpi) gave rise to small masses of microsclerotial initial cells (Figure 1A) that were bright yellow. At 72 hpi (MS2; Figure 1A), developing MS were easily visible, and microsclerotial mass consisting of an accumulation of brown and lightly-pigmented cells appeared on the membrane (Figure 1A). At 96 hpi (MS3; Figure 1A), many pigmented MS were visible and the microsclerotial mass consisted of an accumulation of dark cells across the entire membrane. At 14 dpi (MS4; Figure 1A), mature and darkly pigmented MS were apparent. The individual cells of mature MS also possessed a thickened cell wall surrounded by melanin. In addition to these four stages of MS development, two additional morphological stages of the fungal conidia (CO) and germinating conidia (GC) were sampled for RNA-Seq and comparisons of differential gene expression.

### Overview of the *V. dahliae* transcriptome

The pipeline of RNA-Seq analysis is shown in Figure 1B, RNA libraries derived from the samples were pair-end sequenced using an Illumina high-throughput sequencing platform. After stringent quality checks and tag cleaning, we obtained a total of 158,501,901 reads from the six libraries with an average of 26,416,983 reads in each sample, ranging from 25,596,902 to 27,056,223 reads (Table 1, Additional file 1: Figure S1). The *V. dahliae* (strain VdLs.17) genome assembly and the 10,535 predicted genes of this strain were used as a reference sequence (Broad Institute, Verticillium group Database) [40]. In total, 85.8% of the total reads (135,989,758 reads) from all six samples were aligned to the genome of *V. dahliae*, strain VdLs.17 by Tophat with less than 2 bp mismatches [49]. The total mapped reads represented about 423-fold coverage of the genome and 955-fold coverage of the annotated *V. dahliae* transcriptome. Approximately 14.2% of total reads did not map to the genome, likely reflecting either sequence differences between strains (the smoke tree strain and the lettuce strain VdLs.17), gaps in the current assembly, sequencing errors, or alternative splicing that exists in the reference genome of the Broad Institute.

**Table 1:** **RNA-Seq statistics**

Sample	Insert size (bp)^a^	Total reads^b^	Mapped reads^c^	Perfect match reads^d^	Unmapped reads^e^	Reads mapped to genes^f^
CO	193.341 bp	27056079	23660245 (87.45%^g^)	19767391 (73.06%^g^)	3395834 (12.55%^g^)	15750739 (58.22%^g^/66.57%^h^)
GC	195.521 bp	27056223	23268435 (86.00%)	19630309 (72.55%)	3787788 (14.00%)	15904017 (58.78%/68.35%)
MS1	188.009 bp	26190923	22036154 (84.14%)	18611864 (71.06%)	4154769 (15.86%)	14344871 (54.77%/65.10%)
MS2	194.213 bp	25596902	21727696 (84.88%)	18413763 (71.94%)	3869206 (15.12%)	14297493 (55.86%/65.80%)
MS3	195.993 bp	26410805	22551758 (85.39%)	19100267 (72.32%)	3859047 (14.61%)	14932675 (56.54%/66.22%)
MS4	190.443 bp	26190969	22745470 (86.84%)	19246089 (73.48%)	3445499 (13.16%)	14879393 (56.81%/65.42%)
**Total**		**158501901**	**135989758 (85.80%)**	**114769683 (72.41%)**	**22512143 (14.20%)**	**90109188 (56.85%/66.26%)**

^a^The length of fragments used for sequencing.

^b^The number of reads generated from sequencing after filtering low quality reads (Q ≤ 5).

^c^The number of reads mapped to the reference genome within 2 bp mismatch.

^d^The number of reads mapped to the reference genome with no mismatch.

^e^The number of reads that could not be mapped to the reference genome within 2 bp mismatch

^f^The number of reads mapped to the annotated genes within 2 bp mismatch.

^g^The percentages of reads account for the total reads.

^h^The percentages of reads account for the mapped reads.

Of the mapped reads, 114,769,683 (72.4% of total) reads were perfectly mapped to the *V. dahliae* genome without mismatch. Furthermore, 56.9% of the total reads could be mapped to the annotated genes with less than 2 bp mismatches, indicating that almost 30.0% of the total reads mapped to non-annotated regions, including the intergenic regions or other non-coding regions. The numbers of differentially expressed perfectly matched reads and reads mapped to the annotated genes in each stage were not significantly different (Table 1, Additional file 1: Figure S1). The unmapped percentages of reads were found to be lower (between 12.6% and 15.9%).

**Figure 2:**
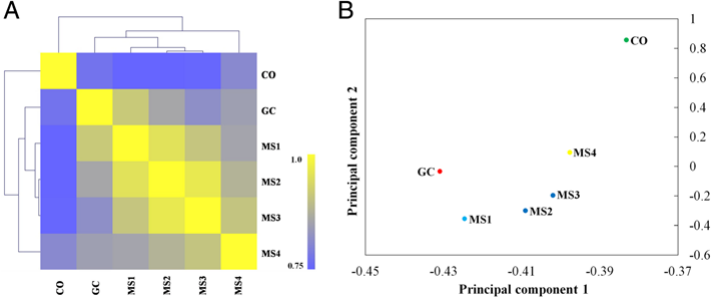
**Correlation in expression patterns among the six RNA-sequenced libraries. A**. Heatmap of the Pearson correlation of RNA-Seq samples according to gene expression level. Clustering was analyzed based on the expression data of 10,158 genes. **B**. Principal component analysis (PCA) of the transcriptomes during microsclerotia formation and germinating conidia. For the principal components 1 and 2, eigenvalues are 86.2% and 6.7%, respectively. The analysis was performed by the MultiExperiment Viewer using the expression data from 10,158 genes.

Gene expression levels were determined by Fragments Per Kilobase of gene per Million mapped fragments (FPKM). Mapping revealed that 10,158 (96.4% of 10,535 total annotated VdLs.17 genes) predicted genes were expressed, ranging from 9,412 (89.3%) to 9,939 (94.3%) among the six libraries (Table 2). Approximately 78.0% (8,222) –87.9% (9,267) of genes were expressed with FPKM >1. Expression patterns identified by RNA-Seq were validated by reverse transcription quantitative real-time PCR (qRT-PCR) analysis of selected genes from each group. Analysis of the RNA-Seq and qRT-PCR data sets revealed correlation between the two (Additional file 2: Figure S2). To examine the correlation of gene expression patterns among the six samples, the Pearson relationships of paired samples were calculated with FPKM of all expressed genes. Samples sharing similar gene expression patterns were clustered together according to the Pearson correlation. This correlation analysis revealed that stages MS1, MS2 and MS3 shared similar expression patterns as stage GC in the larger clade, while the expression patterns in stages MS4 and CO were distinct from each other. Furthermore, the pattern of gene expression in stages MS1-MS4 and GC clustered together, while the stage of CO was in a distinct clade (Figure 2A). Principal component analysis (PCA) was employed to examine the six samples according to their gene expression profiles. Principal components 1 and 2 explained 86.2% and 6.7% of the variance, respectively. Stage CO was distinct from the other five samples. MS1, MS2 and MS3 were clustered together and belonged to a cluster with GC. Stage MS4 was distinct from stages MS1-3 (Figure 2A, 2B). Therefore, the PCA result was consistent with the hierarchical clustering.

**Table 2:** **Distribution of gene expression values among developmental stages examined**

Sample	FPKM^a^ > 0	0 < FPKM < =1	1 < FPKM < =10	10 < FPKM < =100	FPKM > 100
CO	9412 (89.34%^b^)	1190 (12.64%^c^)	2879 (30.59%^c^)	4319 (45.89%^c^)	1024 (10.88%^c^)
GC	9443 (89.63%)	1188 (12.58%)	2388 (25.29%)	4805 (50.88%)	1062 (11.25%)
MS1	9747 (92.52%)	929 (9.53%)	2335 (23.96%)	5314 (54.52%)	1169 (11.99%)
MS2	9858 (93.57%)	757 (7.68%)	2202 (22.34%)	5696 (57.78%)	1202 (12.19%)
MS3	9933 (94.29%)	711 (7.16%)	2199 (22.14%)	5756 (57.95%)	1267 (12.76%)
MS4	9939 (94.34%)	672 (6.76%)	2158 (21.71%)	5743 (57.78%)	1366 (13.74%)

^a^FPKM, fragments per kilobase of transcript per million mapped fragments.

^b^The percentage of genes accounting for all annotated genes in *V. dahliae* (10,535).

^c^The percentage of genes accounting for all expressed genes in each library.

### Clustering analysis reveals enrichment of particular gene categories expressed during MS formation

**Figure 3:**
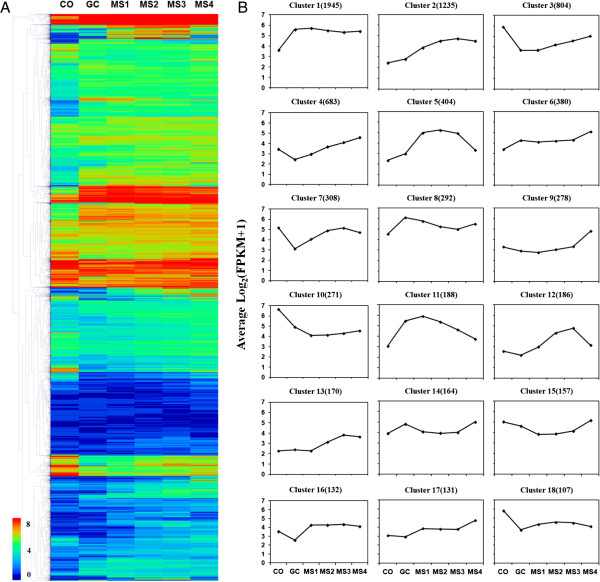
**Clustering of gene expression. A**. The log_2_ of FPKM for each gene was used for hierarchical analysis of the heat map at each of the six selected developmental stages (CO, GC, MS1, MS2, MS3 and MS4). The heat map illustrates that 10,158 expressed genes were classified into 132 gene-expression clusters generated by clustering. The colors correspond to the log_2_ of FPKM values, ranging from bright blue to red. **B**. Log_2_ average gene-expression levels of the top 18 clusters identified. The number of genes in each cluster is bracketed.

To elucidate dynamic changes in the *V. dahliae* transcriptome during MS formation, the clustering affinity search technique (CAST) was employed to generate clusters [50]. CAST analyses of the 10,158 expressed genes revealed 132 clusters, with gene numbers within clusters ranging from 1945 to 1. Most of the clusters had no more than 20 genes. However, each of the top 18 clusters contained more than 100 genes (a range from 1945 to 107), and contained about 77.1% of the total detected genes, illustrating the major gene expression clusters (Figure 3). Increased transcript levels were observed in clusters 1, 2, 5, 6, 11, 12, 13, 16 and 17 in stages MS1-MS4 as compared with stage CO, and comprised 47.0% of the total expressed genes (Figure 3B) whereas genes in clusters 3, 10 and 18 were down-regulated in stages MS1-MS4 compared to stage CO, and comprised 11.6% of the total expressed genes.

According to the gene expression pattern described earlier, and the cluster analysis results, a large variety of genes were differentially expressed during MS formation. To ascribe gene functions to those genes displaying differential expression patterns, gene ontology (GO) enrichment analysis was performed for genes from the top 18 clusters shown in Figure 3B by Blast2GO with Fisher’s Exact Test, and compared with the whole genome background filtered with false discovery rate (FDR) correction (≤0.01). The results of enriched GO terms revealed an overrepresentation of different gene functions in certain clusters. For example, genes encoding protein metabolic processes, such as ribonucleoprotein complex biogenesis, primary metabolic processes, and stress responses, were enriched in cluster 1, which was expressed at increased levels at each stage compared to the CO stage (Figure 3B). The genes in cluster 5 function mainly in signaling pathways, and increased in expression until MS3, at which point there were declining levels of expression (Figure 3B). Genes of cluster 7 were functionally enriched in transport. The functional enrichment of cluster 8 revealed cofactor metabolic processes, oxidation-reduction processes, and chromosome segregation. However, analyses did not reveal functional enrichment in other clusters, such as clusters 2, 4, 9, 10, 12, 13, 16 and 18. The full list of enriched GO terms and corresponding genes are provided in Additional file 3: Table S1.

**Figure 4:**
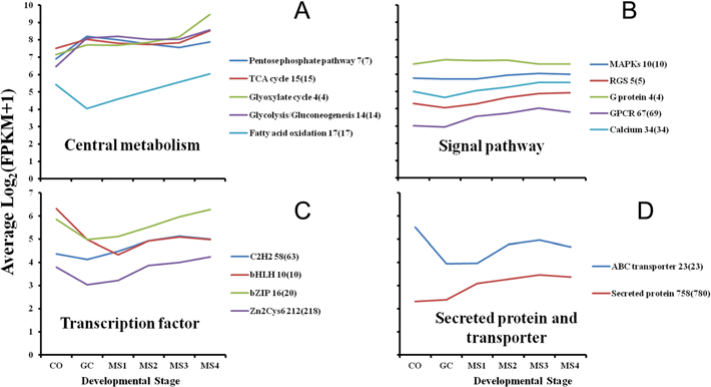
**Global expression analysis shows distinct expression patterns of metabolic processes, signal pathways, transcription factors and secreted proteins during microsclerotia formation. A**. to **D**. Expression trends of genes encoding proteins in four functional categories. **A**. The central metabolic pathways include the glyoxylate cycle, glycolysis/glucogeogenesis, TCA cycle, pentose phosphate pathway, fatty acid oxidation; **B**. Signal transduction and components, such as MAPK cascades, RGSs (regulators of G protein signaling), GPCR (G protein coupled receptors) and calcium signaling. **C**. Four main classes of transcription factors, i.e. C_2_H_2_, bHLH, bZIP and Zn_2_Cys_6_. **D**. Putative secreted proteins and ABC transporter genes. y axes are log_2_-transformed total intensities. The total numbers within a gene family are shown in numerals and the numbers of expressed genes within a family are shown in parentheses.

Several gene families identified in other fungi, including in *V. dahliae*, have established functional roles of importance in growth, development, and pathogenesis. To further understand the expression patterns of these gene families, 16 different families or groups were selected and analyzed further (Figure 4). The results of this analysis illustrated diverse expression patterns, but relative to the GC stage, there was up-regulation of genes encoding products involved in fatty acid oxidation and the glyoxylate cycle (Figure 4A), calcium and GPCR-mediated signaling (Figure 4B), selected classes of transcription factors (Figure 4C), as well as secreted proteins and transporters (Figure 4D) during MS formation (MS1-MS4). As shown, selected branches of energy production pathways including glycolysis/gluconeogensis, TCA cycle, pentose phosphate pathway, fatty acid oxidation and glyoxylate cycle were up-regulated in MS formation compared to the CO stage (Figure 4A), while genes encoding proteins typical of signaling pathways, such as MAPK pathway members were slightly up-regulated during MS formation (Figure 4B, Additional file 4: Figure S3). The genome-wide expression analysis of transcription factors of *V. dahliae* suggested that certain transcription factors were also down-regulated in GC stages and up-regulated in stages MS1-MS4 (Figure 4C). Furthermore, more than 500 genes encoding secreted proteins showed an up-regulated pattern of expression in stages MS1-MS4 (Figure 4D); some of these genes were highly expressed (fold change >50) in MS formation, and 21 of these genes encoded small, cysteine-rich secreted proteins (<=300 aa) (Additional file 5: Figure S4, Additional file 6: Table S2).

### Gene expression profile during MS development

RNA-Seq provides digital readings of gene expression levels [51]. To determine which genes were expressed at each developmental stage, we examined the dynamics of gene expression throughout MS development in the genome-wide transcriptomic data. The distribution of gene expression values was different among the six stages studied. Nearly 12.0% of the expressed genes were detected with low expression values (0 < FPKM ≤ 1) in stages CO and GC, while only 8.0% (average) were detected in stages MS1-MS4, suggesting that many genes expressed with 0 < FPKM ≤1 in CO or GC stages were elevated transcript levels during the MS formation. However, the number of genes with moderate expression values (1 < FPKM ≤100) or high expression values (FPKM >100) in stages MS1-MS4 were increased relative to stages CO or GC, suggesting up-regulation of genes to meet the requirements of MS formation (Table 2). The number of expressed genes with FPKM >1 were gradually increased, from 8,818 (MS1) to 9,267 (MS4) (Table 2).

**Figure 5:**
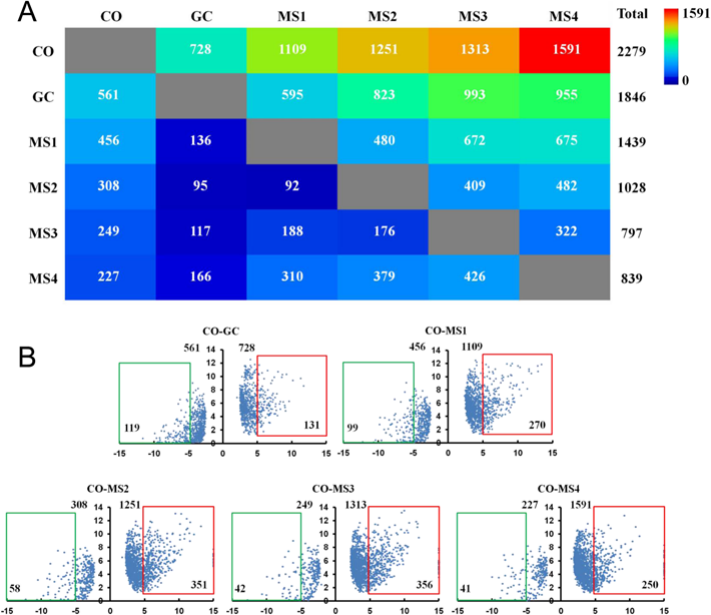
**Significant changes in gene expression and the distribution of differentially expressed genes. A**. The color-coded matrix shows number of significantly up-regulated genes in pairwise comparisons. Total represents the total number of genes significantly up-regulated compared to all other samples. Height, a complete gene significantly up-regulated genes (q value <0.05). **B**. The distribution of log_2_ fold-change and log_2_ expression value of the GC, and MS1-MS4 developmental stages compared to the CO stage. The *y* axes are log_2_ FPKM + 1of expressed genes in GC, and MS1-MS4 stages, and *x* axes are log_2_ fold change of the corresponding expression value compared with that of the CO stage. The numerical value on the top of each chart represents the number of differentially expressed genes, down- (left) or up-regulated (right). The numerical value in the green or red boxes indicates the number of up- or down- regulated genes with >5 fold-change.

To determine which genes were differentially expressed during MS formation, pairwise comparisons were performed using counts of the significantly up- or down-regulated (adjusted p value < 0.05) genes. The number of significantly up-regulated genes was dramatically increased in stages MS1-MS4; however, the number of significantly down-regulated genes was reduced compared with the CO stage (Figure 5A). MS4 represented the stage with the largest number of significantly up-regulated genes and the least number of down-regulated genes compared with the CO stage, consistent with the distribution of gene expression at this stage. We further examined the expression level and log_2_ (fold change) distribution analysis of genes that were significantly differentially expressed at MS1-MS4 and GC stages compared with stage CO (Figure 5B). The number of genes with log_2_ (fold change) > 5 in each stage was consistent with that of significantly up-regulated genes. MS1-MS3 stages consisted of more genes with log_2_ (fold-change) >5 as compared with stage CO (Figure 5B). Using this approach, 600 significantly up-regulated genes and 124 significantly down-regulated genes (adjusted p value < 0.05) were identified (Additional file 7: Figure S5), and these were considered as candidate genes involved in MS development.

GO analysis was performed for the 724 genes significantly differentially expressed in MS formation compared to the CO stage to examine potential molecular mechanisms regulating MS formation. Of the 724 genes, 365 up-regulated genes and 70 down-regulated genes were annotated with GO terms. GO analysis of the up-regulated genes identified processes related to metabolism, catabolism, transport, and the functions related to catalytic, transporter, hydrolase activities (Additional file 7: Figure S5, Additional file 8: Table S3). In addition, GO enrichment analysis was performed with the 600 up-regulated and 124 down-regulated genes compared with the whole genome with p value <0.05. Genes significantly up-regulated represented functional enrichment in structural molecules (GO:0005198), peptidase (GO:0008233), protein kinase (GO:0004672), hydrolase (GO:0016787) activities, and enrichment in processes including cellular carbohydrate metabolism (GO:0044262), cellular protein metabolism (GO:0044267), ribonucleoprotein complex biogenesis (GO:0022613), macromolecule biosynthesis (GO:0009059) and cellular component biogenesis (GO:0044085). On the other hand, the 124 significantly down-regulated genes represented functional enrichment in transporter (GO:0005215) and oxidoreductase (GO:0016491) activities, and in the transport processes (GO:0006810) (Table 3). In conclusion, the 600 up-regulated genes in the MS stages were mainly involved in protein metabolism, carbohydrate metabolism, and biosynthetic processes, including the macromolecule (ribosomal proteins) biosynthesis.

**Table 3:** **GO enrichment terms of significantly regulated genes during MS1-MS4 stages vs stage CO (p value <0.05)**

Terms	FDR^a^	P-value	Expression pattern	No. genes^b^
Ribonucleoprotein complex biogenesis (GO:0022613)	0.089519378	0.002883868	up	23
Cellular carbohydrate metabolic process (GO:0044262)	0.132284495	0.006317295	up	25
Cellular component biogenesis (GO:0044085)	0.132284495	0.007286858	up	26
Cellular protein metabolic process(GO:0044267)	0.203232906	0.017429065	up	51
Generation of precursor metabolites and energy (GO:0006091)	0.203232906	0.017982239	up	14
Gene expression (GO:0010467)	0.203232906	0.01894544	up	24
Structural molecule activity (GO:0005198)	3.28E-04	1.39E-06	up	25
Peptidase activity (GO:0008233)	0.022419783	1.90E-04	up	27
Motor activity (GO:0003774)	0.233071632	0.022714608	up	4
Oxidoreductase activity (GO:0016491)	0.263043145	0.02675015	up	64
Protein kinase activity (GO:0004672)	0.332984226	0.038095653	up	15
Hydrolase activity (GO:0016787)	0.351323453	0.041682444	up	88
Transport (GO:0006810)	0.587336379	0.012443567	down	19
Transporter activity (GO:0005215)	0.418960736	0.001775257	down	13
Oxidoreductase activity (GO:0016491)	0.454356181	0.003850476	down	20

^a^FDR**,** False discovery rate, adjusted p-value of the test statistics.

^b^Number of genes enrichment in each GO terms among the significantly regulated genes.

### Differential expression of ubiquitin-dependent protein catabolism and cell death-associated genes during MS development

Some of the MS cells undergo autolysis or death during MS formation in *V. dahliae*[8]. According to the GO categories and enrichment analysis, there were more than twenty genes involved in protein metabolic processes, most of which participated in proteolysis. Among the genes involved in protein metabolic processes, five were involved in proteasome formation, and included *VDAG_00111* (proteasome subunit alpha type 6), *VDAG_08991* (proteasome component pup2), *VDAG_02924* (proteasome component pre3 precursor), *VDAG_03131* (proteasome component pup3), *VDAG_04256* (proteasome subunit beta type 7 precursor) (Additional file 9: Figure S6A). Genes encoding products involved in protein modification processes were also enhanced during MS formation, such as ubiquitination, important for proteasome-mediated degradation of proteins [52].

**Figure 6:**
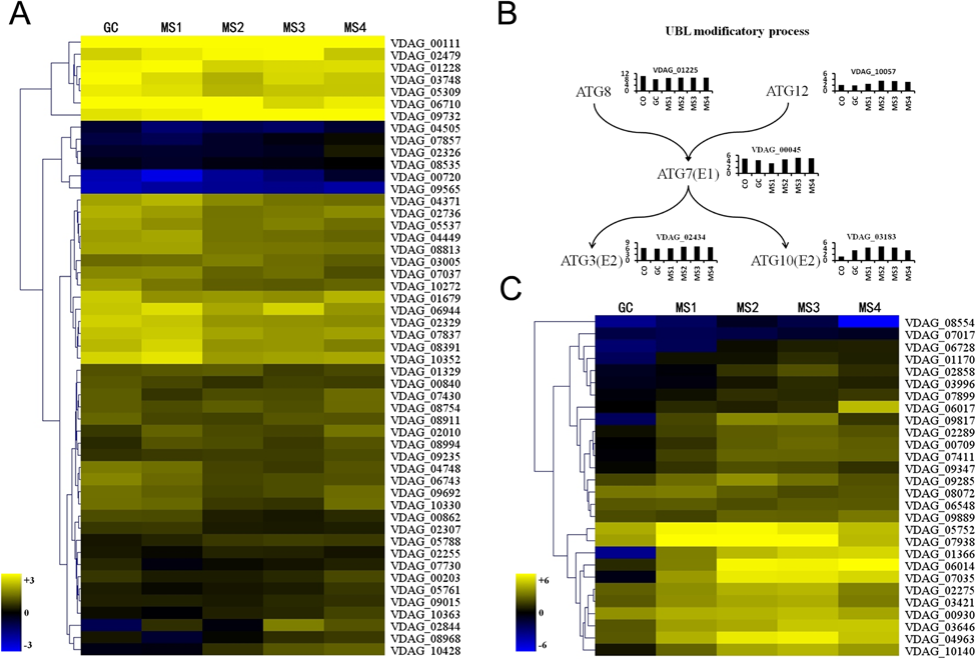
**Ubiquitin-dependent protein catabolism and cell death. A**. The heatmap shows levels of transcription abundance from genes involved in ubiquitin-dependent protein catabolism during microsclerotia formation (stages MS1-MS4). Levels of expression are represented as the log_2_ ratio of transcript abundance as compared to the CO stage. Genes showing similar expression patterns have been clustered. **B**. Two ubiquitin-like protein (UBL) modification pathways in *V. dahliae* and expression levels of genes encoding UBL proteins. For each protein, the bar graph shows abundance of transcripts encoding this protein in CO, GC and microsclerotia formation (MS1-MS4, black bars left to right). **C**. Heatmap of genes encoding a heterokaryon incompatibility protein (HET) domain.

We identified 51 genes involved in ubiquitin-dependent protein catabolic processes in *V. dahliae* using homology searches (Figure 6A). Expression profiles of the ubiquitin-dependent genes showed that almost 90.0% of these genes were up-regulated during MS formation (Figure 6A), suggesting that ubiquitination and subsequent protein degradation might play an important role in MS formation. The autophagy-related genes *ATG3*, *ATG7*, *ATG8*, *ATG10* and *ATG12* are involved in ubiquitin-like protein (UBL) degradation processes [52, 53]. Orthologous genes were identified in *V. dahliae*, such as *VDAG_02434* (ATG3), *VDAG_00045* (ATG7), *VDAG_01225* (ATG8), *VDAG_03183* (ATG10), *VDAG_10057* (ATG12). Figure 6B shows the expression patterns in which the genes of the left branch of the UBL protein degradation process showed small changes in expression during MS formation, while those genes on the right branch showed relatively large expression changes, especially *VDAG_03183*. These results suggest that the proteolysis process was induced during MS formation, potentially contributing to protein degradation during MS formation.

Other genes involved in autophagy and cell death in *V. dahliae* were identified. In total, 23 autophagy genes were identified in *V. dahliae*, which were then divided into two groups, nonselective autophagy and selective autophagy [54]. Expression profiles of these genes showed stable expression patterns across developmental stages examined, except *VDAG_03183* (nonselective) and *VDAG_01208* (selective), which were up-regulated during MS formation (Additional file 9: Figure S6B). In addition, some genes encoding the Het domain were up-regulated during MS formation in *V. dahliae*, although over 90.0% of the Het-related genes were neither up- nor down-regulated during MS formation (Figure 6C). Vegetative incompatibility is an apoptotic-type heterokaryon incompatibility, and proteins containing a Het domain mediate the apoptotic-type pathway [55–59]. Potentially, cell death observed during MS formation in *V. dahliae*[11] is regulated through mechanisms that also mediate vegetative incompatibility.

### Analyses of lineage-specific gene expression during MS development

**Figure 7:**
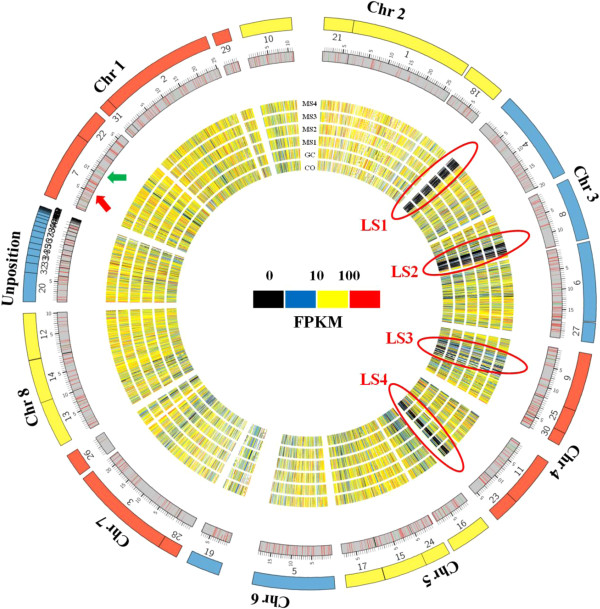
**Global view of gene expression in hyphae, conidia, germinating conidia, and microsclerotial formation stages MS1-4.** Seven concentric circles from inside to outside represent fungal stages CO, HY, GC, MS1, MS2, MS3 andMS4, respectively. Color representation (from black to red) illustrates the expression level (FPKM, fragments per kilobase of transcripts per million mapped fragments) of each transcript in each sample. The red and green colored bars in the gray circle represent genes significantly up- or down-regulated during microsclerotia development compared to the CO stage (q value <0.05), respectively. Each chromosome is shown by the number of the first outer circle, which represents the *V. dahliae* strain VdLs.17 scaffold number assigned by the Broad Institute. Red ovals represent the four LS regions in the *V. dahliae*, marked as LS1, 2, 3, 4. Numbers under the outer circle correspond to the respective supercontigs of the *V. dahliae* genome.

There exist four lineage specific (LS) regions in *V. dahliae* strain VdLs.17, and these regions are of interest since they encode 354 predicted protein-encoding genes that play important roles such as secondary metabolism, transcription, and pathotype specificity [40]. To reveal changes in the expression of genes located in LS regions in the transcriptome during MS formation, the global gene expression profile in the *V. dahliae* genome in the six samples was visualized by Circos [60]. The genome-wide analyses of gene expression indicated that the majority of genes were expressed with FPKM values ranging from 10 to 100, and the expressed genes were uniformly distributed in the *V. dahliae* genome (Figure 7). However, the non-expressed genes were mainly distributed in the supercontig 4, supercontig 8 and supercontig 9 on Chr 3, and supercontig 23 on Chr 4 in all the six stages (Figure 7, red circles). Interestingly, the four LS regions in VdLs.17 are located within these particular supercontigs (Figure 7, red circles).

Transcription factors encoded within the LS regions potentially regulate gene expression of not only LS-associated genes, but also other core genes in the genome of *V. dahliae* that have roles in MS development. We followed the expression patterns of bZIP transcription factors to ascertain a potential role of these genes in regulating development in *V. dahliae*. Among 20 bZIP transcription factor-encoding genes of *V. dahliae*, strain VdLs.17, five genes (*VDAG_02348*, *VDAG_02408*, *VDAG_02411*, *VDAG_02415* and *VDAG_09148*) are located within the LS regions and were separated in a distinct clade with other bZIP transcription factors in a phylogenetic analysis (Additional file 10: Figure S7), similar to those results previously reported [40] All five homologs of these LS-encoded genes, with the exception of *VDAG_09148*, were not differentially expressed during MS formation in the smoke tree strain of *V. dahliae*. Furthermore these genes are Verticillium-specific (Additional file 11: Figure S8).

### Expression profiles of genes involved in carbohydrate metabolism and melanin biosynthesis during MS formation

Genes involved in carbohydrate metabolic process were significantly up-regulated during the MS formation based on GO annotation analysis. To further study the dynamics of hexose metabolism during MS formation, glycolysis/gluconeogenesis, tricarboxylic acid, pentose phosphate and glyoxylate pathways were examined (Figure 4A, Additional file 6: Table S2). Expression profiles showed that genes controlling hexose metabolism pathways were differentially expressed during MS formation (Additional file 12: Figure S9). For example, two key enzyme encoding genes *VDAG_04087* (hexokinase) and *VDAG_01206* (pyruvate kinase) in glycolysis were significantly up-regulated during MS1-MS4 stages, indicative of an up-regulation of glycolysis during MS formation. Conversely, two genes that are critical for gluconeogensis (*VADG_07446* and *VDAG_10101*) were down-regulated.

Acetyl-CoA is an important intermediate product of metabolism and plays a very important role in the cell’s energy requirement, metabolic pathways, and appressorium formation in rice blast disease [39]. RNA-Seq analyses of genes expressed in MS formation revealed up-regulation of four genes in strain XS11 of *V. dahliae* (corresponding to *VDAG_08164*, *VDAG_09433*, *VDAG_06356* and *VDAG_01642*) that are candidate genes involved in catalyzing the pyruvate to generate acetyl-CoA, suggesting an increase in the amount of acetyl-CoA during MS formation.

**Figure 8:**
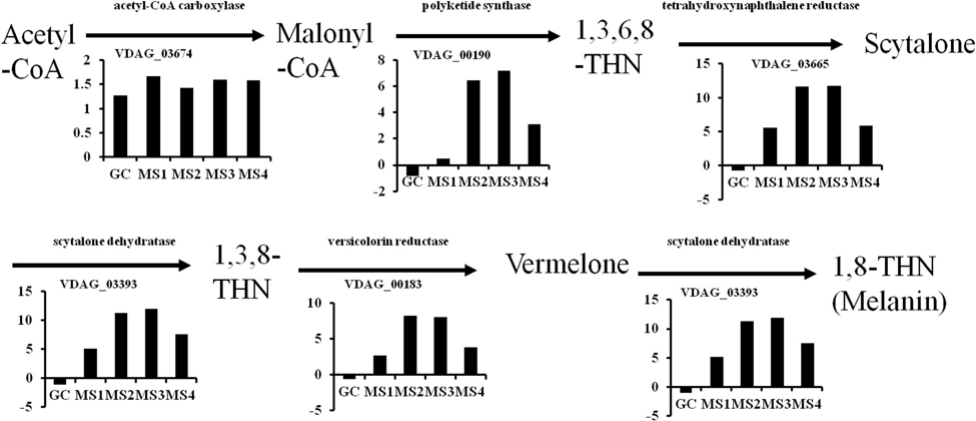
**RNA-Seq reveals significantly high transcript abundance of genes involved in melanin biosynthesis.** The diagram shows the process of fungal melanin biosynthesis as described by Soanes et al. [39]. For each enzyme, the histograms show the abundance of transcripts encoding enzymes in melanin biosynthesis, gene expression levels calculated by log_2_ FPKM of transcript abundances in stages GC, MS1, MS2, MS3 and MS4 compared with the CO stage.

The polyketide pathway is one of the most important metabolic processes involved in the growth, development, and pathogenicity in filamentous fungi. Melanin is a prevalent polyketide in pathogenic fungi, and melanin biosynthesis is well studied in pathogenic fungi [61, 62]. Genes involved in melanin biosynthesis were identified in *V. dahliae* by homology searches, including polyketide synthase (*VDAG_00190*), scytalone dehydratase (*VDAG_03393*) and hydroxynaphthalene reductase (*VDAG_03665*). Expression profiles of these and other genes encoding enzymes of the melanin biosynthesis pathway are shown in Figure 8. *VDAG_00190*, *VDAG_03393* and *VDAG_00183* homologs in strain XS11 were all highly expressed and significantly up-regulated (over 10-fold change) during the MS formation as compared with stage CO (Figure 8). The expression profile was consistent with phenotypic observations of MS formation, during which the accumulation of melanin was dramatically increased at stages MS3 and MS4 (Figure 1A). Decreases in the expression levels of *VDAG_03674*, *VDAG_00190*, *VDAG_03393* and *VDAG_00183* were observed in MS4 as compared to MS3 in strain XS11, at the completion of melanin deposition in the MS (Figure 8).

### Discovery of alternative splicing

**Figure 9:**
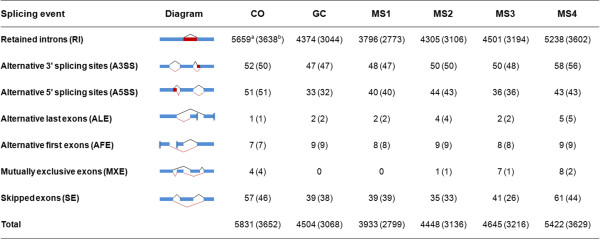
**Statistics of alternative splicing events. a**, represents number of alternative splicing events in each stage. **b**, represents number of genes that undergo alternative splicing events in each stage in parentheses.

Alternative splicing (AS) for the creation of new gene transcripts can increase regulatory complexity and control of developmental programs in higher organisms. A software SplicingViewer [63] was employed to identify putative AS events in the six different developmental stages of *V. dahliae* that were examined in this study. This analysis tool enabled detection of potential splicing junctions and identification of seven putative types of AS events, such as skipped exons (SE), mutually exclusive exons (MXE), alternative 5′ splicing sites (A5SS), alternative 3′ splicing sites (A3SS), alternative first exons (AFE), alternative last exons (ALE) and retained intron (RI) [64]. More than 50.0% (5,212) expressed genes were detected in the existing AS events representing 9,167 AS events in the six stages examined (Figure 9, Additional file 13: Table S4). Four primary types of AS events, namely, retained intron (RI), skipped exons (SE), alternative 5′ splicing sites (A5SS), alternative 3′ splicing sites (A3SS) were common in *V. dahliae*. The other three types of AS events were sporadic, and mutually exclusive exons (MXE) were rarely observed in *V. dahliae*. Furthermore, 44.8% (2,337) of the AS genes produced more than two isoforms (Additional file 14: Table S5). More than ten alternative transcripts were identified for a single gene, such as the *VDAG_07349* homolog, encoding a cysteine-rich-protein that contains 14 exons. There were 31 different isoforms of *VDAG_07349* that were observed, as concluded from all the six developmental stages examined. Over 95.0% of the AS events were RI in all stages, and the other AS events consisted of less than 1.0%, respectively (Figure 9), suggesting that the RI was the most common event observed in *V. dahliae*. We performed RT-PCR to validate the RI events of three genes, i.e. for homologs of *VDAG_02669*, *VDAG_03183*, *VDAG_03665*. All three genes exhibited alternatively spliced transcripts, consistent with the results of RNA-Seq (Additional file 15: Figure S10).

## Discussion

The formation of MS in *V. dahliae* is critically important for survival and propagation of this fungus, representing a significant developmental event in the disease cycle. The long term survival of the MS in soil poses a major obstacle for effective control of Verticillium wilts through cultural practices such as crop rotation. The structure of the MS, including a thickened cell wall and heavy melanin deposition, protects the pathogen from various environmental assaults and even fungicidal activity [5]. This study was undertaken to begin elucidating of the genetics and biochemical processes underpinning MS development, which may yield insight into control strategies to combat vascular wilt disease of smoke trees, and other plant hosts of economic or aesthetic importance.

In this work, RNA-Seq was performed on samples from six developmentally distinct stages, ranging from conidia (CO) and germinating conidia (GC) through four stages of MS formation and maturation (MS1 to MS4). Due to the actual difficulties of obtaining MS from real condition such as in plant or soil, especially during MS development, we obtained MS in vitro. The rationale for this approach was to define MS-associated gene expression during MS formation. Analyses of MS formation on an artificial surface ensured that gene expression data were exclusively from the fungus, rather than the smoke tree host. It is also technically difficult to sample different developmental stages of MS in plant tissues and soil. Likewise, MJ Neumann and KF Dobinson [42] employed a method to collect MS samples for studies of gene expression during MS development in *V. dahlia*e using EST analyses from *in vitro* cultures. Given the limitations of EST-based analyses and advancements in NGS technologies, the dynamics of transcriptome expression during MS formation were studied using the relatively new NGS technology of RNA-Seq. This technology enabled identification of metabolic pathways of interest, as well as alternative splicing. Analyses of these transcriptome data revealed that this was an effective approach, providing an extensive catalog of expression values over the range of six distinct developmental stages. Moreover, of the 158.5 million sequenced reads, 114.8 million reads uniquely mapped to the genome without mismatch, and 90.1 million reads mapped perfectly to the annotated genes of the *V. dahliae* strain VdLs.17 sequence.

The increased expression of particular genes observed in this study was expected; including those involved in melanin biosynthesis, or selected genes that were previously identified as important in MS formation. Melanin biosynthetic genes in *V. dahliae* were expressed at relatively high levels during MS formation consistent with the culture phenotype (increased pigmentation) observed. In addition, *VDH1* (*VDAG_02273*), a hydrophobin protein-encoding gene, is involved in MS formation, and *VDH1* is specifically expressed in developing microsclerotia [12, 45]. In concurrence with the results of that study, the expression level of *VDH1* was significantly increased in the stages of MS1-MS3, especially in MS1 and MS2 (Additional file 16: Table S6). The finding of the developmentally regulated expression of *VDH1* in this study may also explain the lack of differential expression of *VDH1* observed in RNA-Seq analyses of gene expression in which only 10 day MS-producing cultures and 10 day cultures not forming MS were compared [43]. In the study of Duressa et al. [43] stages equivalent to MS1 or MS2 were not analyzed.

Analyses of GO functional category enrichment indicated that carbohydrate and protein metabolic processes, and ribonucleoprotein biosynthetic processes were significantly enriched among up-regulated genes, while transport processes were significantly enriched among down-regulated genes. Functional classification revealed that peptidase, protein kinase, and hydrolase activities were significantly up-regulated during MS1-MS4 stages. Among the carbohydrate metabolic processes, several genes involved in energy production were enriched; such as *VDAG_04087* (hexokinase), *VDAG_01206* (pyruvate kinase), and *VDAG_03029* (enolase). These results suggested that these differentially expressed genes are involved in a broad range of physiological functions, especially in proteolysis, protein modification, during MS formation. Enrichment of peptidase activity may also accompany processes associated with cell death or autolysis in MS formation [11]. On the other hand, most genes encoding plant cell wall-degrading enzymes, such as the polysaccharide lyase and carbohydrate esterase gene families, showed low expression levels during MS formation. The carbohydrate-binding module 1 gene family, which is significantly enriched in *V. dahliae* relative to most other fungi [40], also showed low expression at the MS developmental stages analyzed in this study.

Stage-specific differences were extensive in *V. dahliae* at the gene regulatory level during MS development. Among the genes annotated in *V. dahliae*, strain VdLs.17, dozens of genes that were differentially expressed during MS formation were identified (Additional file 7: Figure S5). This indicated that MS-stage-specific gene regulation occurs at the transcriptome level during MS formation. These data provided a basis for further assessing the roles of individual genes, especially those MS-specific genes potentially involved in cellular differentiation and MS formation.

The four major lineage-specific (LS) regions identified in the genome of strain VdLs.17 of *V. dahliae* are thought to contribute to adaptation to different host niches [40] and encode virulence factors [41]. While the total LS region contents differ between strains of *V. dahliae*[41], a total of 354 genes are located in the four LS regions of strain VdLs.17; and some of these genes encode proteins involved in lipid metabolism, plant-fungal interactions, and transcriptional regulation [40]. Interestingly, analyses of three EST libraries produced under low nutrient or complete medium revealed the LS genes of VdLs.17 were significantly up-regulated compared to genes in the genome core sequence. Furthermore, recent work indicates enrichment for *in planta*-expressed LS genes in the *V. dahliae* strain JR2 [41]. In this study, the majority of genes in LS regions of *V. dahliae* were either not expressed or showed low expression in MS1 to MS4, suggesting that genes of the LS regions are suppressed at the developmental stages examined relative to those of the genome core, and also may be influenced by the differing gene content of LS regions between different strains examined. Consistent with this hypothesis, expression of *VDAG_02354*, encoding a high osmolarity glycerol response (HOG1) protein, and located in LS region 1, was not detected in all six developmental stages examined in this study. The other copy of the *HOG1* gene (*VDAG_08982* was identified outside of the LS regions in the genome of VdLs.17 and expressed at a higher level during MS formation. The deletion of the HOG1 gene (*VDAG_08982*) from the smoke tree strain resulted in significantly reduced and delayed production and developmental progress of MS in vitro (Xiao et al., unpublished data). Additional analyses revealed that genes encoding LS region-associated bZIP transcription factors were not expressed, or showed low expression in the stages of MS formation, while other bZIP transcription factors located in non-LS regions were expressed during MS formation.

The RNA-Seq analysis provided useful information on alternatively spliced transcripts in *V. dahliae*. These analyses are critical for studies that aim to assess gene regulation and gene function in MS development and in other developmental processes. The number of genes undergoing AS were mainly estimated based on overlapping introns, which does not take intron retention and splice junctions into account [65]. Over 95.0% AS events were due to intron retention. As in other fungi, such as *Aspergillus oryzae* and *Ustilago maydis*, RI is the predominant form of AS [66, 67]. A total of 5,212 genes underwent AS events, while there was an average of 3,250 genes that underwent AS events in each stage. This suggests that stage-specific genes undergo AS at different times during MS development. Further screens of these genes are required to increase understanding the role of AS in the development and pathogenesis in the smoke tree wilt fungus. Assembly of transcription from short sequencing reads remains a computational challenge. Therefore, it is essential to validate the predicted AS events by laboratory and field experiments.

In summary, an RNA-Seq strategy was employed to gain insight on the biology and molecular basis of MS development of smoke tree vascular wilt fungus, *V. dahliae*. Functional categories of genes such as those involved in carbohydrate metabolism, proteolysis, and cell death were differentially regulated during MS formation. Comprehensive, high-resolution gene expression maps enabled detection of a large number of AS events that provide a key resource for further studies that aim to understand the molecular underpinnings of MS development and other developmental processes in this fungus. Further, de novo assembly of transcripts from RNA-Seq data represents a potential avenue for gene annotation [68–71]. The analysis of splicing events detected herein may shed light on alternate splicing in *V. dahliae*, and help to understand the roles of AS in MS formation and other developmental processes.

## Conclusion

In this study, we have conducted a RNA-Seq analysis of the MS developmental process in smoke tree wilt fungus *V. dahliae* XS11. A global view of gene expression profiles and a large-scale stage-specific transcriptome alterations during MS development are revealed. Further analysis show that genes involved in glycolytic pathway, melanin biosynthesis and protein catabolism are dramatically up-regulated in MS stages. In addition, a large number of AS events are detected among CO, GC and MS stages. Our results provides a key resource for understanding the biological and molecular basis of MS development of *V. dahliae*.

## Methods

### Fungal strain and growth conditions

*Verticillium dahliae* strain XS11, which was single spore-isolated from a smoke tree in Fragrant Hills Park, Beijing, was used in these experiments. Cultures were initially grown on PDA (potato dextrose agar). Conidia were harvested from cultures grown in liquid CM, as previously described [72]. Conidia were collected by filtering through two layers of Miracloth (Calbiochem, USA), and the conidial suspension was sedimented by low speed (4000 rpm) centrifugation. The conidia were cultured at a concentration 10^5^ conidia/ml for germination in the liquid basal medium (BM, 10 g/L glucose, 0.2 g/L sodium nitrate, 0.52 g/L KCl, 0.52 g/L MgSO_4_^.^7H_2_O, 1.52 g/L KH_2_PO_4_, 3 μM thiamine HCl, 0.1 μM biotin, 15 g/L agar, kindly provided by Dr. Katherine Dobinson, Agriculture and Agri-Food Canada, London, Canada) by shaking at 30 rpm for 12 hours at 24°C. Germinating conidia were harvested similarly.

### Microsclerotial developmental stages

To observe the developmental process of MS formation of *V. dahliae* in stages MS1-MS4, a cellulose membrane (Ø =80 mm; pore size = 0.22 μm) was placed on BM agar and a suspension of 10^5^ conidia/ml of *V. dahliae* strain XS11 was spread over the cellulose membrane, and incubated in the dark at 24°C. Developmental stages were observed under light microscopy (DM2500, Leica) at 12 hour intervals after incubation on the membrane until 7 days post incubation (dpi). After 7 dpi, the observations were conducted every 2 days for 1 week.

### RNA extraction and validation of expression by RT-PCR and qRT-PCR

Total RNA was extracted from conidia, germinating conidia, and the MS1-MS4 stages of MS formation by using TRIzol Reagent (Invitrogen) and purified with the RNA Mini Kit (Ambion) according to the manufacturer’s instructions. All samples were ground to a fine powder with a mortar and pestle in liquid nitrogen. Total RNA was eluted in RNase-free water and stored at −80°C until further use. For each sample, two biological replicates were used for library preparations. The integrity and quantity of RNA was determined using a Qubit fluorometer (Invitrogen), agarose electrophoresis and the Agilent Bioanalyzer 2100 (Additional file 17: Table S7).

The RNA samples for RT-PCR were incubated at 37°C for 30 min with DNase I (RNase-free) (TaKaRa) to remove DNA contamination before reverse transcription. The mRNA were enriched using Oligo DT, then were transcribed to cDNA using SuperScript III Reverse Transcriptase (Invitrogen). The qRT-PCR was carried out using SYBR green (SuperReal Premix Plus; TIANGEN, China) methodology and the ABI 7500 real-time PCR system (Applied Biosystems, USA). The *V. dahliae* β-tublin gene was used as internal reference for all the qPCR analyses. Analyses of each gene were conducted in quadruplicate. Relative gene expression was calculated according to the ΔΔCT method. The primers used are described in Additional file 18: Table S8.

### Library preparation for RNA-Seq

Libraries were prepared using RNA-Seq sample preparation kit from Illumina and poly(A) mRNA was enriched from total RNA using oligo (dT) beads. Fragmentation buffer was added for breaking mRNA to short fragments. Using these fragments as template, first-strand cDNA was synthesized by reverse transcription with a random hexamer primer. The second-strand cDNA was synthesized using buffer, dNTPs, RNase H and DNA polymerase I according to kit manufacturer instructions.. Short fragments were purified with QiaQuick PCR extraction kit (Qiagen) and resolved with EB buffer. Sequencing adaptors were added and amplified with PCR. Agarose gel electrophoresis was used to select the fragments with about 200 bp in size. Finally, the libraries were sequenced on Illumina HiSeq™ 2000 (Beijing Genomics Institute, Shenzhen) to produce 90 bp paired-end reads.

### Mapping reads to the *V. dahliae* reference genome

Low quality (Q ≤5) reads containing adapters were removed from the raw reads, and Tophat software (version 2.0.0) [49] was used to align the filtered reads to the published reference genome of *V. dahliae* strain VdLs.17 (http://www.broadinstitute.org/annotation/genome/verticillium_dahliae/MultiHome.html) and to predict exon splice sites allowing less than two mismatches. The pipeline of RNA-Seq analysis is shown in Figure 1B. Total mapped reads were obtained with the parameters “-G (*Vd*) -r 20 --segment-length 30” provided by Tophat and perfect mapped reads were obtained using the parameters “-G (*Vd*) –r 20 --segment-length 30 –read-mismatches 0” provided by Tophat. In addition, reads only mapped to the annotated genes of *V .dahliae* were carried out with the parameters “-T -G (*Vd*) –r 20 --segment-length 30”.

### Transcript assembly and genes expression analysis

Cufflinks software (version1.30) [73] was used to assemble the individual transcripts from RNA-Seq reads which had been aligned to the genome of *V. dahliae,* strain VdLs.17, with Tophat. Gene expression level was calculated using FPKM (fragments per kilobase of transcript per million mapped fragments) in Cufflinks. Due to their low reliability for assembly purposes, Cufflinks filtered low abundance transcripts using the default parameter. Cuffmerge, a component of Cufflinks, was used to merge the transcripts of several samples. Cuffdiff, a package of Cufflinks, was used to identify differentially expressed genes among samples (p value ≤0.05), and the CummeRbund R package [74] was used to visualize differentially expressed genes. Fold changes in gene expression were calculated with log_2_ FPKM compared with that of the control sample, CO. A MultiExperiment Viewer [50] was used to visualize changes in gene expression, cluster analyses, PCA analyses and CAST analyses with threshold affinity value 0.9 and other default parameters. The global views of gene expression patterns were visualized by Circos [60]. Pearson correlation coefficient was calculated among the six samples according to genes’ expression profiles. Venn diagram was drawn through the interactive tool of VENNY [75].

### Gene ontology and functional annotation

Gene Ontology was identified in the GO database through Blast2GO [76] software using the *in silico* translated sequence and default parameters. In addition, functional annotation, classification, and enrichment analysis were performed by Blast2GO software with the default parameters.

### Alternative splicing analysis

Alternative splicing events were identified by SplicingViewer [63]. First, reads were aligned to the reference genome by Bwa allowing less than two mismatches [77]. Second, Samtool was used to obtain unmapped reads from the first step [78], to detect the splice junctions, and obtain the splice junction sequences. Unmapped reads were mapped to the splice junction sequences by Bwa with less than two mismatches. Seven types of alternative splicing events were identified by AlternativeSplicing.jar (http://bioinformatics.zj.cn/splicingviewer/index.php).

### Phylogenetic analysis

Amino acid sequences were aligned using Clustal X (version 1.83) without masking unreliable aligned positions [79]. Phylogenetic trees were constructed using Mega 5.0 by Maximum Likelihood method with at least 1000 bootstrap replications [80].

### Availability of supporting data

RNA-Seq data were submitted to the NCBI SRA database (http://www.ncbi.nlm.nih.gov/Traces/sra/) with the accession number: SRR1232601, SRR1232602, SRR1232630, SRR1232631, SRR1232632, SRR1232674, respectively.

## Electronic supplementary material

Additional file 1: Figure S1: Overview of the RNA-Seq data. All of the developmental stages share similar sets of mapped reads. The number of reads produced from the Illumina platform (blue bar); the number of reads mapped to the reference genome within 2 bp mismatch (red bar); the number of reads perfectly mapped to the reference genome (green bar); the number of reads mapped to the annotated genes (purple bar). (PNG 62 KB)

Additional file 2: Figure S2: Validation of RNA-Seq expression patterns. The RT-qPCR results of the selected genes show similar expression patterns to those detected by RNA-Seq. (PNG 68 KB)

Additional file 3: Table S1: GO enrichment analysis of genes in 18 clusters (p_value < 0.01). (XLSX 12 KB)

Additional file 4: Figure S3: Expression profiles of MAPK cascades. Heatmap showed that four types of MAPK cascades during MS formation. Expression pattern of genes encoding high osmolarity (HOG1) pathway were clustered together. The non-LS HOG1-MAPK maintained the highest expression value compared to other MAPKs. (PNG 685 KB)

Additional file 5: Figure S4: Expression profiles of small secreted proteins (length ≤300aa). Expression profiles of those genes encoding small secreted proteins with at least 50-fold up-regulated during MS formation compared with the CO stage. The levels of expression represent log_2_ FPKM Value +1. * represents genes with cysteine residues <4. (PNG 317 KB)

Additional file 6: Table S2: List of genes involved in metabolism, signal pathway and identified transcription factor. (XLSX 161 KB)

Additional file 7: Figure S5: Functional categorization of genes differentially expressed (up- or down-regulated) during microsclerotia development. (A, C, E) Intersection of MS1-4 stages revealed 600 significantly up-regulated genes vs CO stage, and functional categorization of these genes. (B, D, F) Intersection of MS1-4 stages revealed 124 significantly down-regulated genes vs CO stage, and functional categorization of these genes. (PNG 584 KB)

Additional file 8: Table S3: Significantly regulated genes during microsclerotia formation vs CO stage (p_value < 0.05). (XLSX 46 KB)

Additional file 9: Figure S6: Genes involved in protein metabolic processes and autophagy. A. Genes involved in protein metabolic processes by functional categorization; genes labeled with asterisks are the subunits associated with proteasome formation. B. Heatmap representation of genes involved in autophagy processes. Heatmap shows levels of transcripts abundance; relative levels of expression are presented by moderated log_2_ ratio of transcript abundance in MS developmental stages relative to the CO stage. (PNG 747 KB)

Additional file 10: Figure S7: Phylogenetic analysis and expression profile of bZIP transcription factors of *V. dahliae.* The full-length amino acid sequences of bZIP transcription factors of *V. dahlia*e strain VdLs.17 were aligned using Clustal X and the phylogenetic tree was constructed using Mega 5.0 using the maximum-likelihood method with 1000 replicates. Domain structures are drawn to represent their relative positions. The black solid line represents the corresponding protein and its length. The different-colored boxes represent different domains and their positions in each protein predicted by Sanger Pfam program (http://pfam.xfam.org/) and the yellow ovals represent bZIP domains. The red box indicates an independent cluster of bZIP transcription factors genes, which contain two specific motifs represented at the bottom. The right panel represents heat maps showing the expression pattern of bZIP transcription factors of *V. dahliae*. (PNG 522 KB)

Additional file 11: Figure S8: Phylogenetic analysis of bZIP transcription factors of *V. dahliae* and other fungi. The amino acid sequences of bZIP transcriptional factors of *V. dahliae* strain VdLs.17, and other fungi were aligned using Clustal X and the phylogenetic tree was constructed using Mega 5.0 using the maximum-likelihood method with 1000 replicates. Fungal species are Sc, *Saccharomyces cerevisiae*; Vd, *Verticillium dahliae*; Va, *Verticillium alfalfae* (formerly *V. albo-atrum*); Fv, *Fusarium verticillioides;* Fo, *Fusarium oxysporum*; Fg, *Fusarium graminearum*; Mg, *Magnaporthe oryzae*; Nc, *Neurospora crassa.* Pa, *Podospora anserine*; Hj, *Hypocrea jecorina*. (PDF 42 KB)

Additional file 12: Figure S9: Expression patterns of genes involved in glycometabolism. Genes involved in carbohydrate metabolism including Glycolysis/Gluconeogensis, Glyoxylate cycle, TCA cycle, Pentose phosphate pathway. Heatmap shows levels of transcripts abundance; Level of expression are presented by moderated log_2_ ratio of transcript abundance vs CO stage. (PNG 556 KB)

Additional file 13: Table S4: List of genes undergoing AS events. (XLSX 575 KB)

Additional file 14: Table S5: Statistics of the AS events genes. (XLSX 9 KB)

Additional file 15: Figure S10: RT-PCR validation of three genes undergo RI events. Primers (black arrows) were designed spanning intron region. gDNA represents genomic DNA. cDNA represents complementary DNA. (PNG 163 KB)

Additional file 16: Table S6: Gene expression value of all annotated genes. (XLSX 804 KB)

Additional file 17: Table S7: Quality of Total RNA by Agilent 2000. (XLSX 11 KB)

Additional file 18: Table S8: RT-PCR primers used in this study. (XLSX 11 KB)

## Abbreviations

NGS: Next generation sequencing
CO: Conidia
GC: Conidia germination
MS1: Microsclerotia formation 60 h
MS2: Microsclerotia formation 72 h
MS3: Microsclerotia formation 96 h
MS4: Microsclerotia formation 14d
CM: Complete medium
GO: Gene Ontology
dpi: days post incubation
FPKM: Fragments per kilobase of transcript per million mapped fragments
UBL: Ubiquitin-like protein
CWDEs: Cell wall degrading enzymes
ESTs: Expressed sequence tags
ORF: Open reading frame
MAPK: Mitogen-activated protein kinase
*HOG1*: High osmolarity glycerol
GPCR: G protein–coupled receptor
RGS: Regulator of G-protein signaling
SE: Skipped exon
MXE: Mutually exclusive exon
A5SS: Alternative 5′ splicing site
A3SS: Alternative 3′ splicing site
AFE: Alternative first exon
ALE: Alternative last exon
RI: Retained intron.
