# Supplementary figures and images for: Deep mRNA sequencing reveals stage-specific transcriptome alterations during microsclerotia development in the smoke tree vascular wilt pathogen, Verticillium dahliae
Source: BMC Genomics. 2014 May 1;15(1):324. doi: 10.1186/1471-2164-15-324 (PMC4035056; doi:10.1186/1471-2164-15-324)

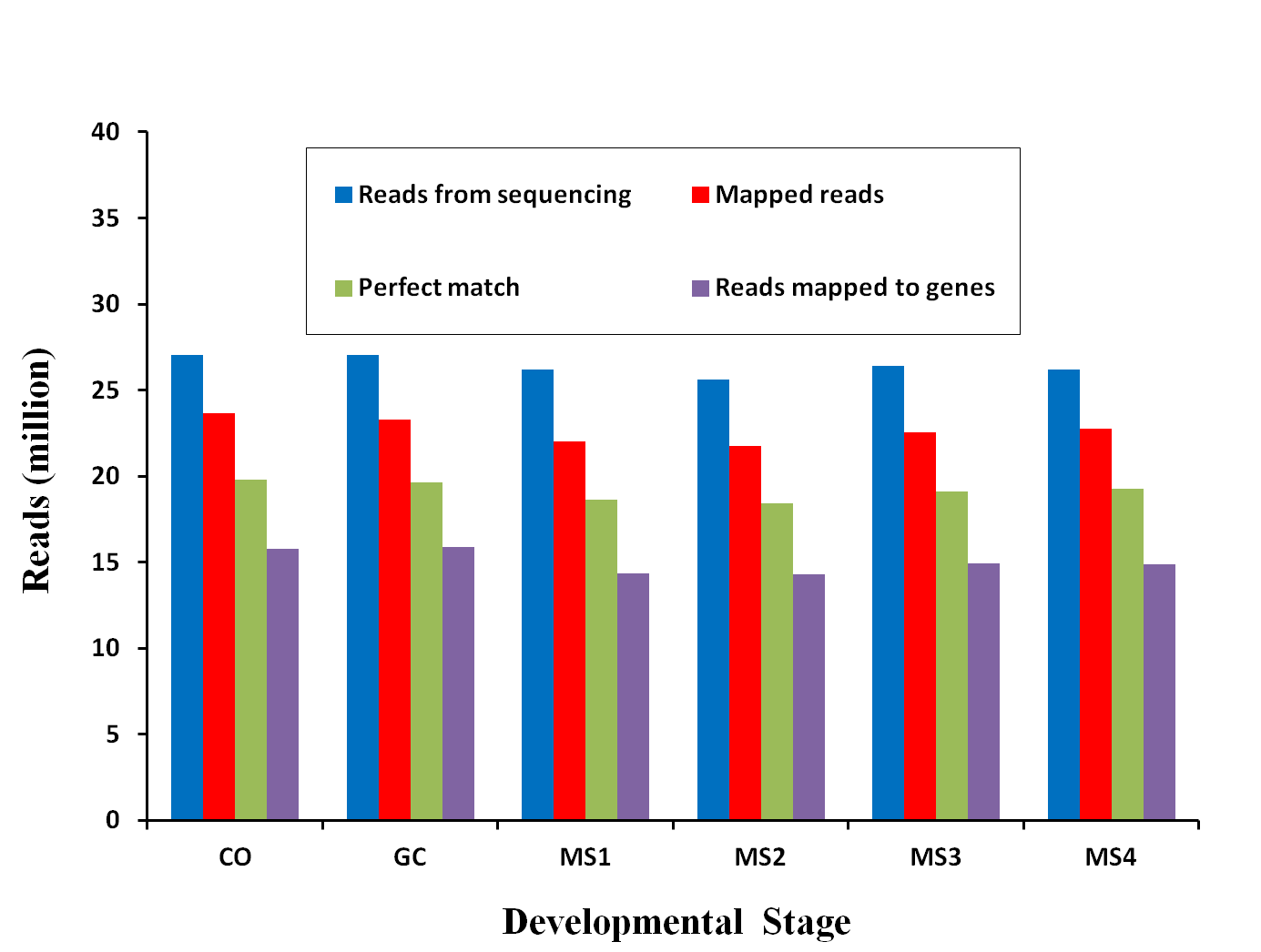

Supplement: Supplementary file 1 — Additional file 1: Figure S1: Overview of the RNA-Seq data. All of the developmental stages share similar sets of mapped reads. The number of reads produced from the Illumina platform (blue bar); the number of reads mapped to the reference genome within 2 bp mismatch (red bar); the number of reads perfectly mapped to the reference genome (green bar); the number of reads mapped to the annotated genes (purple bar). (PNG 62 KB) [file 12864_2013_6023_MOESM1_ESM.png]

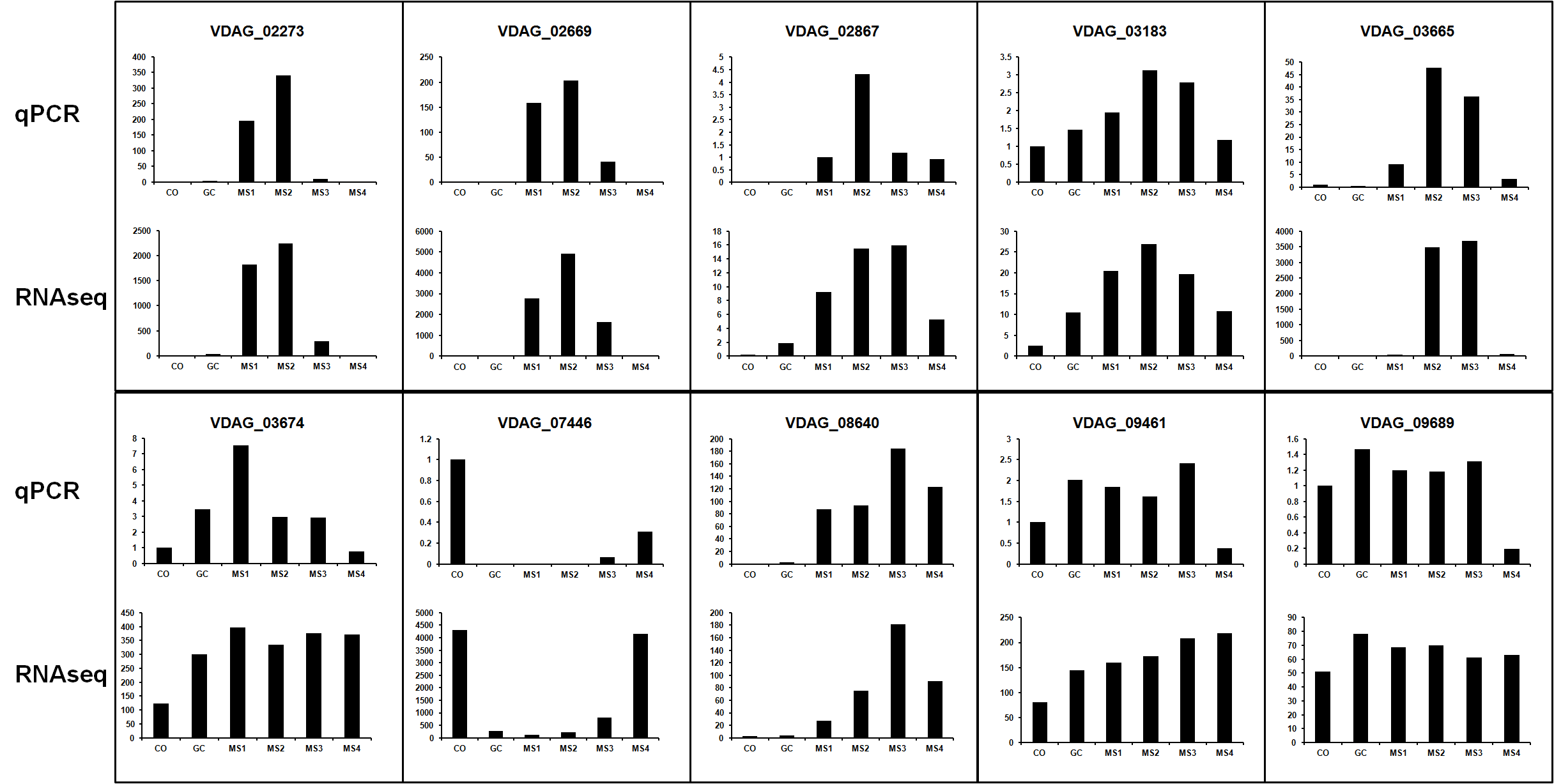

Supplement: Supplementary file 2 — Additional file 2: Figure S2: Validation of RNA-Seq expression patterns. The RT-qPCR results of the selected genes show similar expression patterns to those detected by RNA-Seq. (PNG 68 KB) [file 12864_2013_6023_MOESM2_ESM.png]

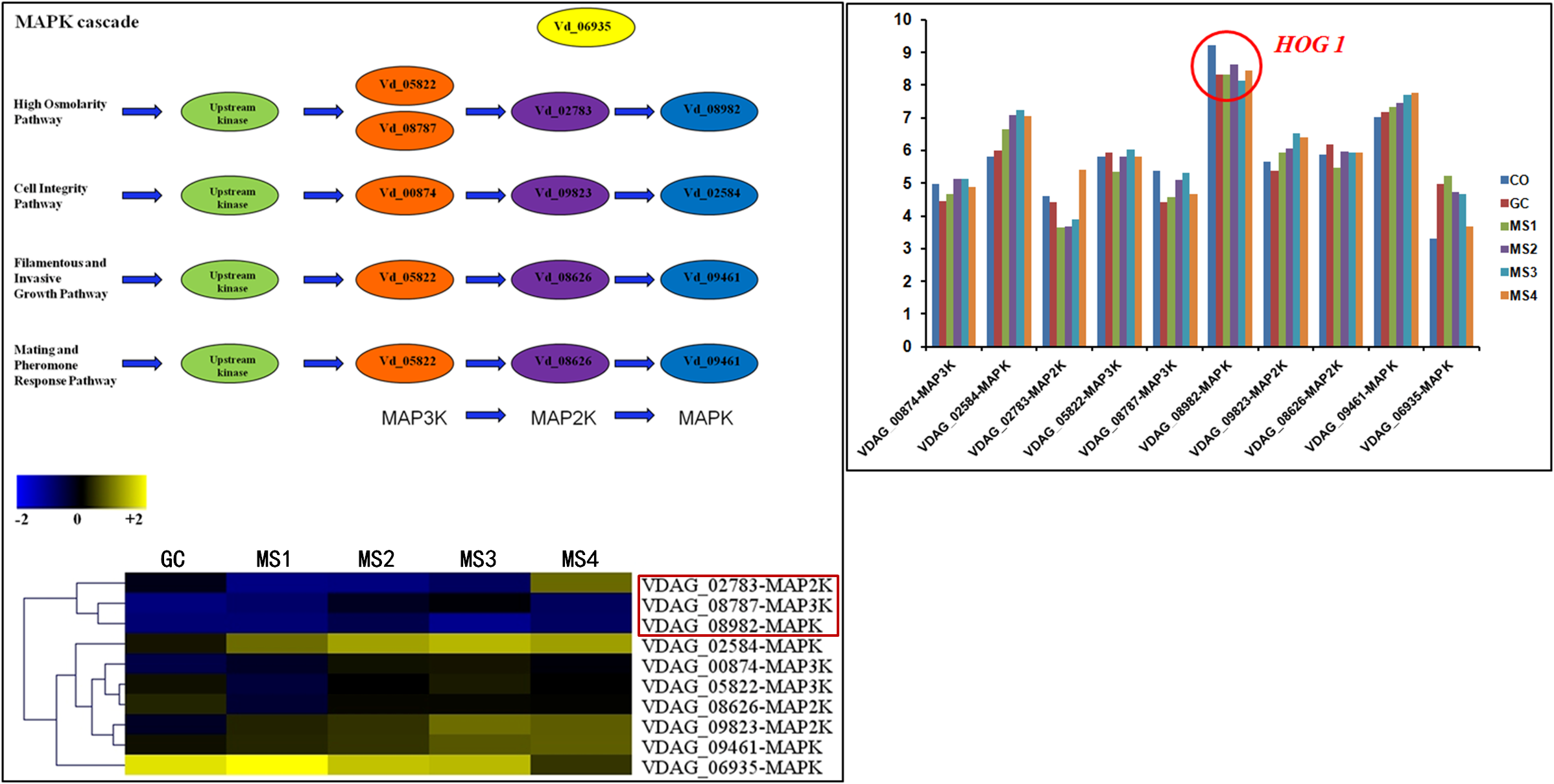

Supplement: Supplementary file 4 — Additional file 4: Figure S3: Expression profiles of MAPK cascades. Heatmap showed that four types of MAPK cascades during MS formation. Expression pattern of genes encoding high osmolarity (HOG1) pathway were clustered together. The non-LS HOG1-MAPK maintained the highest expression value compared to other MAPKs. (PNG 685 KB) [file 12864_2013_6023_MOESM4_ESM.png]

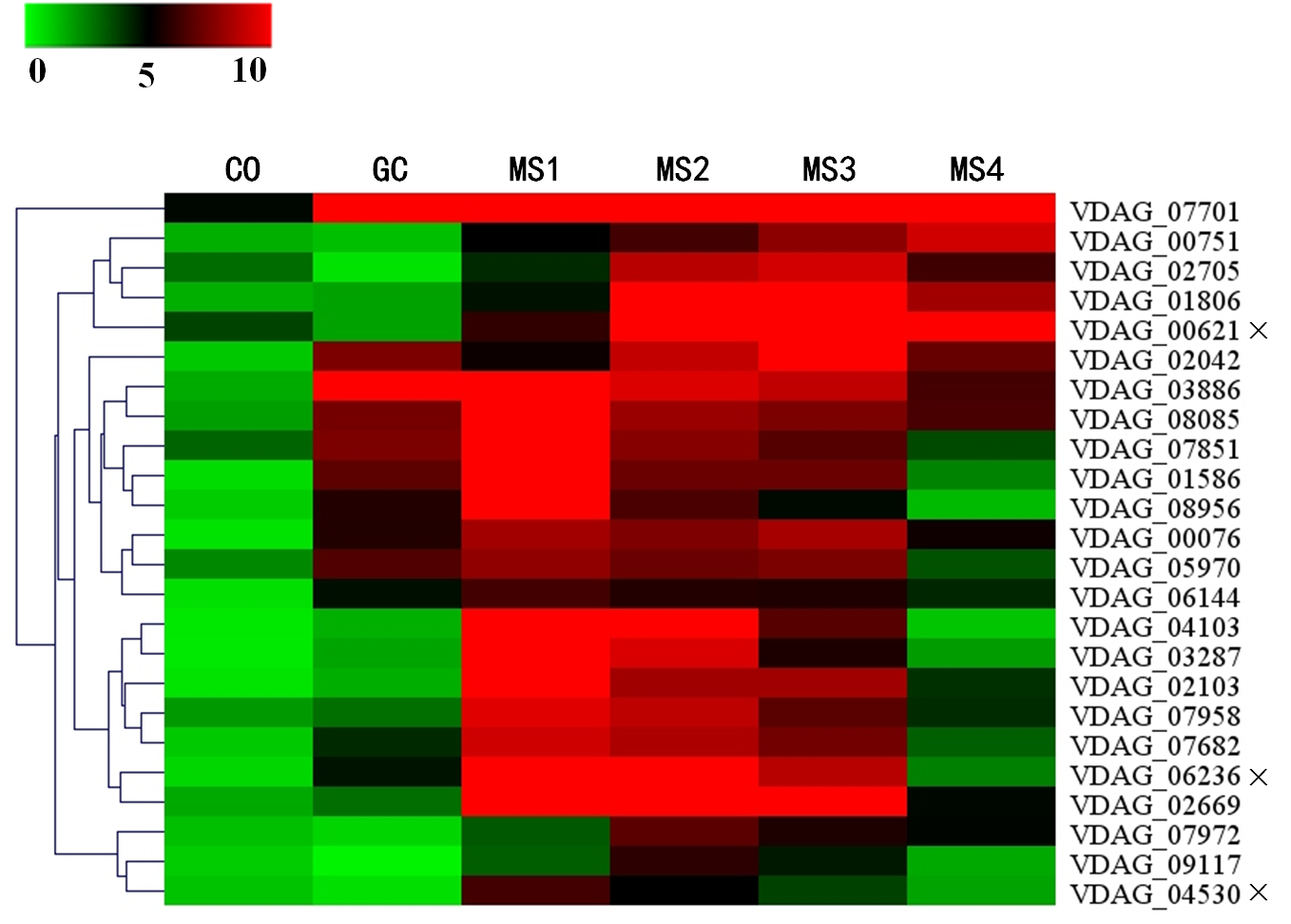

Supplement: Supplementary file 5 — Additional file 5: Figure S4: Expression profiles of small secreted proteins (length ≤300aa). Expression profiles of those genes encoding small secreted proteins with at least 50-fold up-regulated during MS formation compared with the CO stage. The levels of expression represent log2 FPKM Value +1. * represents genes with cysteine residues <4. (PNG 317 KB) [file 12864_2013_6023_MOESM5_ESM.png]

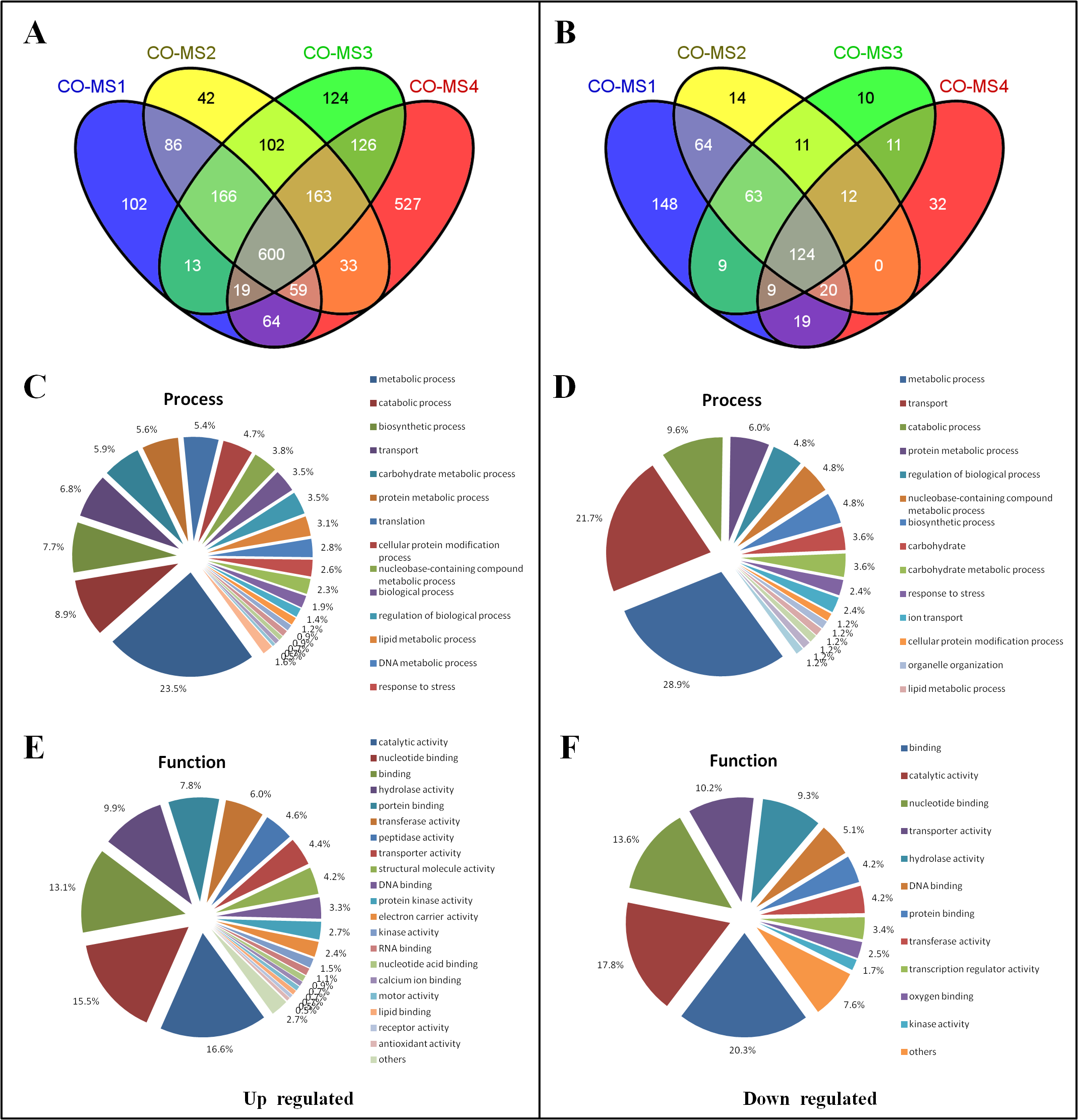

Supplement: Supplementary file 7 — Additional file 7: Figure S5: Functional categorization of genes differentially expressed (up- or down-regulated) during microsclerotia development. (A, C, E) Intersection of MS1-4 stages revealed 600 significantly up-regulated genes vs CO stage, and functional categorization of these genes. (B, D, F) Intersection of MS1-4 stages revealed 124 significantly down-regulated genes vs CO stage, and functional categorization of these genes. (PNG 584 KB) [file 12864_2013_6023_MOESM7_ESM.png]

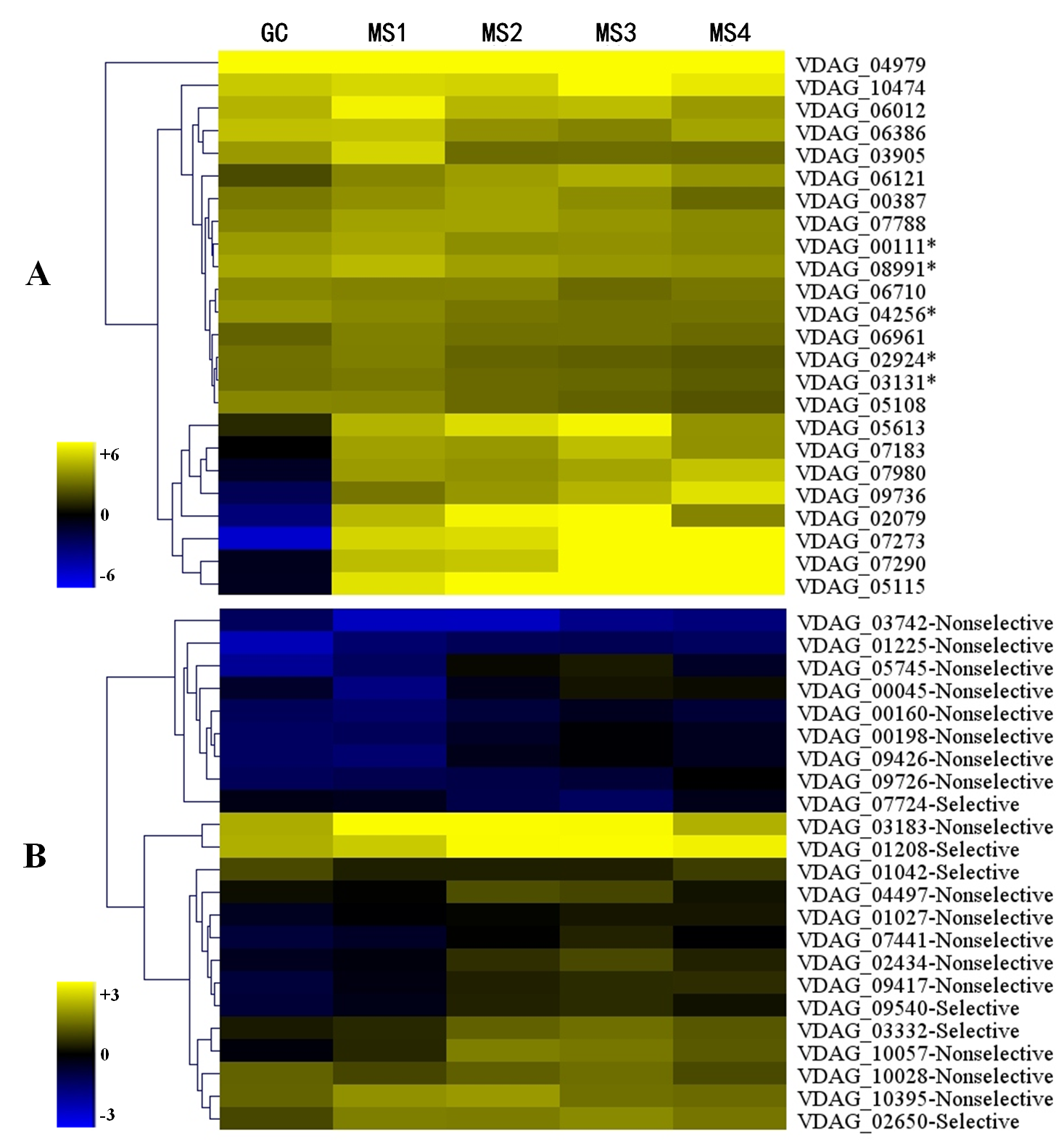

Supplement: Supplementary file 9 — Additional file 9: Figure S6: Genes involved in protein metabolic processes and autophagy. A. Genes involved in protein metabolic processes by functional categorization; genes labeled with asterisks are the subunits associated with proteasome formation. B. Heatmap representation of genes involved in autophagy processes. Heatmap shows levels of transcripts abundance; relative levels of expression are presented by moderated log2 ratio of transcript abundance in MS developmental stages relative to the CO stage. (PNG 747 KB) [file 12864_2013_6023_MOESM9_ESM.png]

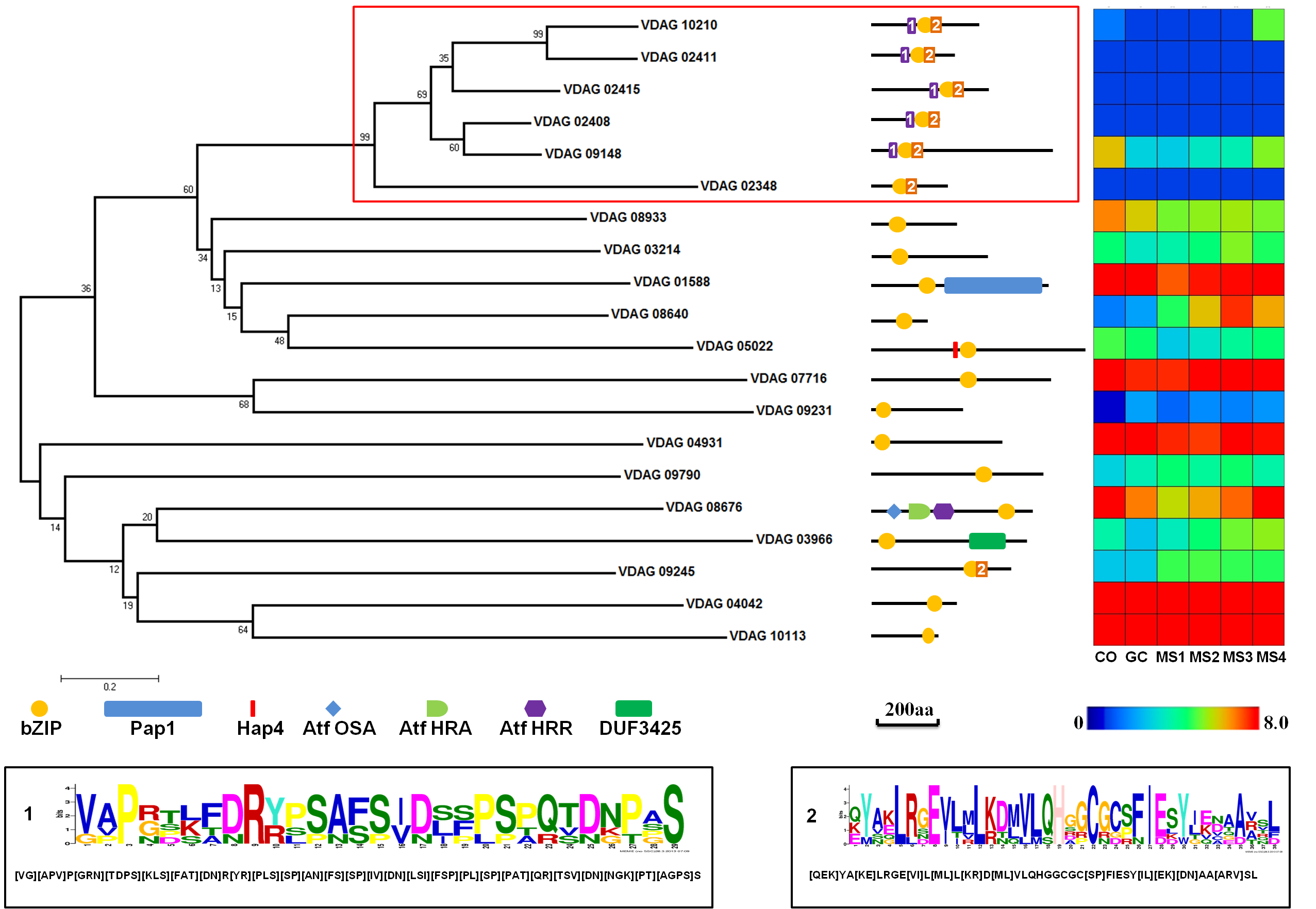

Supplement: Supplementary file 10 — Additional file 10: Figure S7: Phylogenetic analysis and expression profile of bZIP transcription factors of V. dahliae. The full-length amino acid sequences of bZIP transcription factors of V. dahliae strain VdLs.17 were aligned using Clustal X and the phylogenetic tree was constructed using Mega 5.0 using the maximum-likelihood method with 1000 replicates. Domain structures are drawn to represent their relative positions. The black solid line represents the corresponding protein and its length. The different-colored boxes represent different domains and their positions in each protein predicted by Sanger Pfam program (http://pfam.xfam.org/) and the yellow ovals represent bZIP domains. The red box indicates an independent cluster of bZIP transcription factors genes, which contain two specific motifs represented at the bottom. The right panel represents heat maps showing the expression pattern of bZIP transcription factors of V. dahliae. (PNG 522 KB) [file 12864_2013_6023_MOESM10_ESM.png]

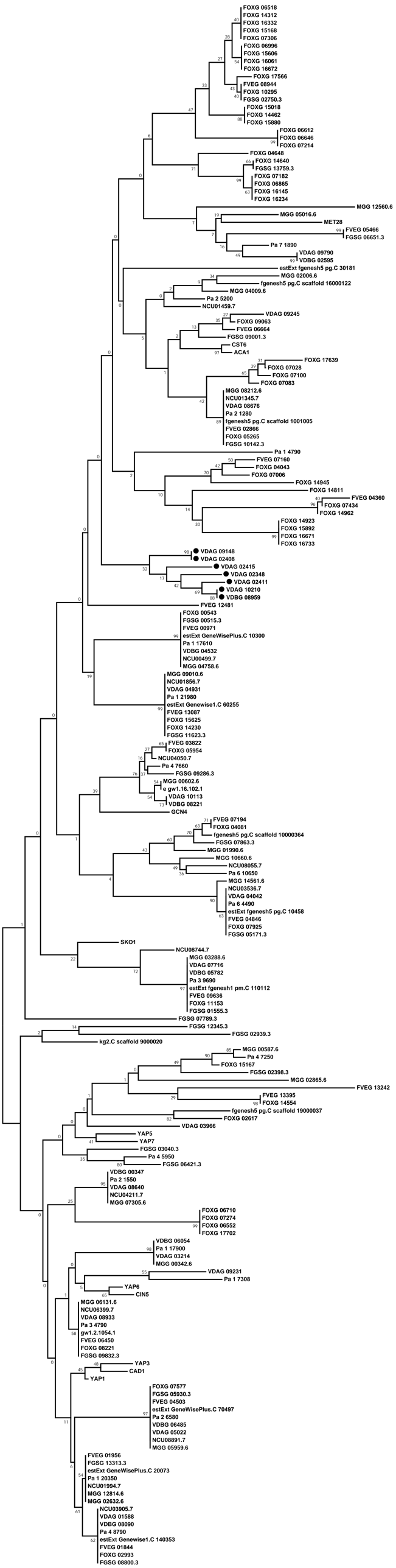

Supplement: Supplementary file 11 — Additional file 11: Figure S8: Phylogenetic analysis of bZIP transcription factors of V. dahliae and other fungi. The amino acid sequences of bZIP transcriptional factors of V. dahliae strain VdLs.17, and other fungi were aligned using Clustal X and the phylogenetic tree was constructed using Mega 5.0 using the maximum-likelihood method with 1000 replicates. Fungal species are Sc, Saccharomyces cerevisiae; Vd, Verticillium dahliae; Va, Verticillium alfalfae (formerly V. albo-atrum); Fv, Fusarium verticillioides; Fo, Fusarium oxysporum; Fg, Fusarium graminearum; Mg, Magnaporthe oryzae; Nc, Neurospora crassa. Pa, Podospora anserine; Hj, Hypocrea jecorina. (PDF 42 KB) [file 12864_2013_6023_MOESM11_ESM.pdf]

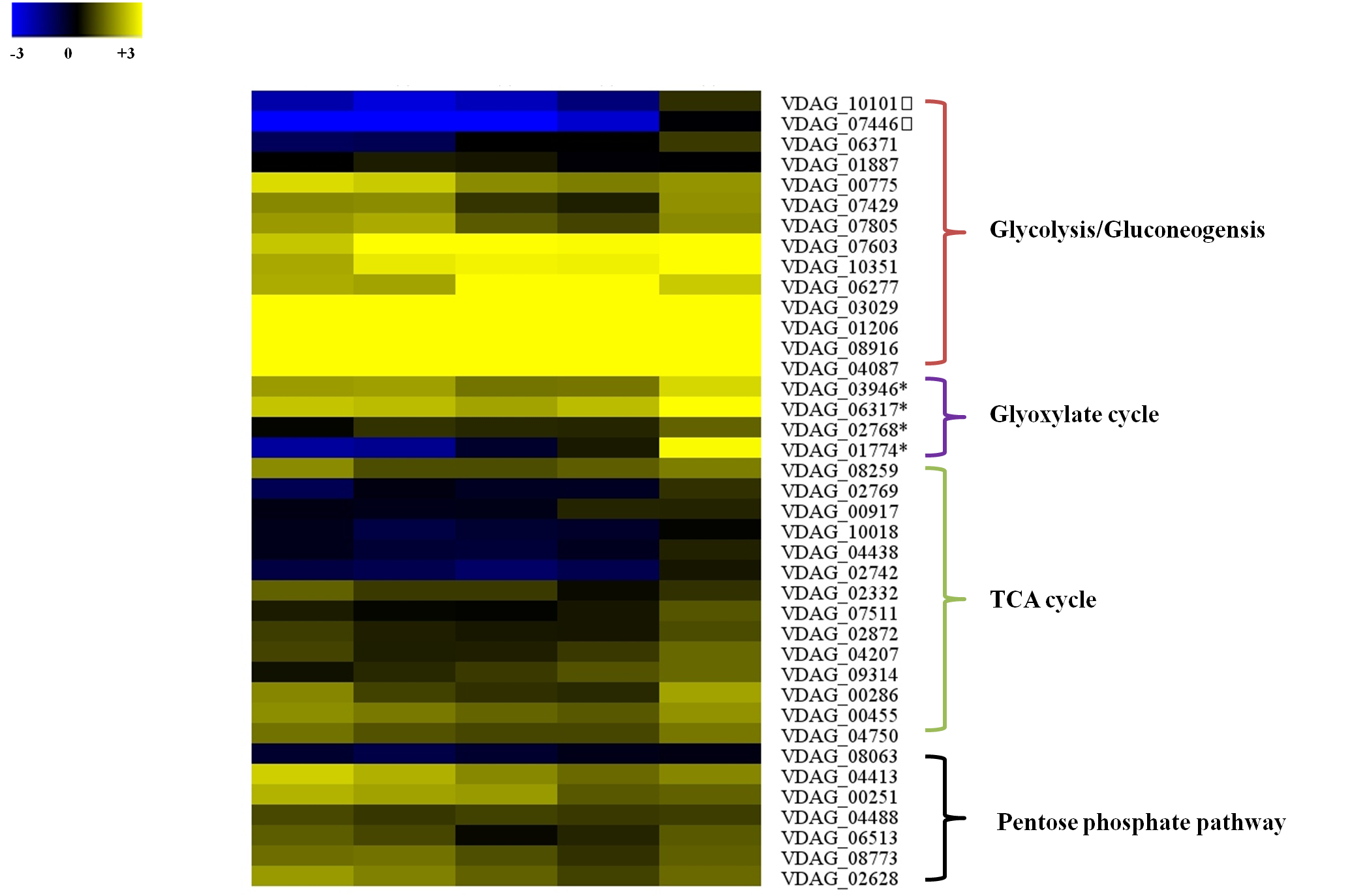

Supplement: Supplementary file 12 — Additional file 12: Figure S9: Expression patterns of genes involved in glycometabolism. Genes involved in carbohydrate metabolism including Glycolysis/Gluconeogensis, Glyoxylate cycle, TCA cycle, Pentose phosphate pathway. Heatmap shows levels of transcripts abundance; Level of expression are presented by moderated log2 ratio of transcript abundance vs CO stage. (PNG 556 KB) [file 12864_2013_6023_MOESM12_ESM.png]

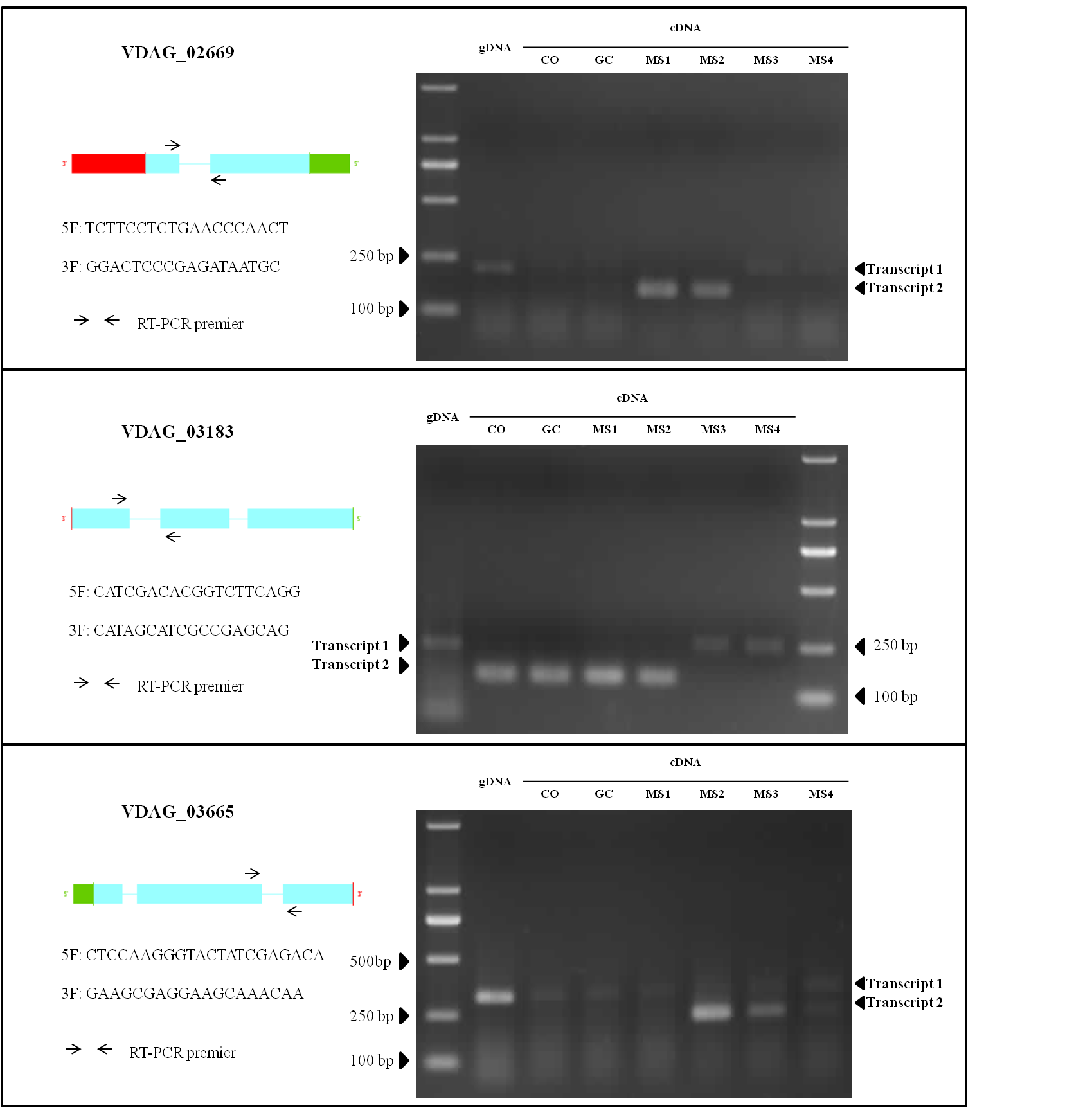

Supplement: Supplementary file 15 — Additional file 15: Figure S10: RT-PCR validation of three genes undergo RI events. Primers (black arrows) were designed spanning intron region. gDNA represents genomic DNA. cDNA represents complementary DNA. (PNG 163 KB) [file 12864_2013_6023_MOESM15_ESM.png]
